# Natural products modulate cell apoptosis: a promising way for treating endometrial cancer

**DOI:** 10.3389/fphar.2023.1209412

**Published:** 2023-06-08

**Authors:** Xin Zhou, Yiwei Zeng, Runchen Zheng, Yuemei Wang, Tao Li, Shanshan Song, Su Zhang, Jinzhu Huang, Yulan Ren

**Affiliations:** ^1^ School of Acupuncture-Moxibustion and Tuina, Chengdu University of Traditional Chinese Medicine, Chengdu, China; ^2^ School of Chinese Classics, Chengdu University of Traditional Chinese Medicine, Chengdu, China; ^3^ School of Nursing, Chengdu University of Traditional Chinese Medicine, Chengdu, China; ^4^ Department of Gynecology, School of Clinical Medicine, Chengdu University of Traditional Chinese Medicine, Chengdu, China

**Keywords:** natural products, endometrial cancer, apoptosis, signal pathway, anti-cancer effects

## Abstract

Endometrial cancer (EC) is a prevalent epithelial malignancy in the uterine corpus’s endometrium and myometrium. Regulating apoptosis of endometrial cancer cells has been a promising approach for treating EC. Recent *in-vitro* and *in-vivo* studies show that numerous extracts and monomers from natural products have pro-apoptotic properties in EC. Therefore, we have reviewed the current studies regarding natural products in modulating the apoptosis of EC cells and summarized their potential mechanisms. The potential signaling pathways include the mitochondria-dependent apoptotic pathway, endoplasmic reticulum stress (ERS) mediated apoptotic pathway, the mitogen-activated protein kinase (MAPK) mediated apoptotic pathway, NF-κB-mediated apoptotic pathway, PI3K/AKT/mTOR mediated apoptotic pathway, the p21-mediated apoptotic pathway, and other reported pathways. This review focuses on the importance of natural products in treating EC and provides a foundation for developing natural products-based anti-EC agents.

## 1 Introduction

Endometrial cancer (EC) refers to a prevalent epithelial malignancy occurring in the endometrium and myometrium of the uterine corpus. It is the most common gynecological malignancy in developed countries and the second most common in developing countries (Sung et al., 2021; Akazawa and Hashimoto, 2022). The morbidity of EC is estimated to increase by more than 50% worldwide by 2040 (Moore and Brewer, 2017; Zhang S. et al., 2019; Brooks et al., 2019). EC typically occurs in postmenopausal women, while a rising incidence is observed in the premenopausal population due to the increasing onset of obesity globally (Moore and Brewer, 2017). Conventional treatments for EC include surgical resection, radiotherapy, chemotherapy, and hormonotherapy, depending on the cancer stage (An et al., 2021). Though these treatment regimens benefit the patients, the outcomes and prognosis of those at the advanced and recurrent stage or with metastasis remain poor (Lu and Broaddus, 2020).

On the other hand, these options are often accompanied by adverse consequences. For example, a hysterectomy is recommended for patients with higher-grade EC or myometrium invasion, while these patients have to lose their childbearing ability (Lu and Broaddus, 2020). For patients receiving chemotherapy, the issue of drug resistance would not be ignored, which could compromise the therapeutic effects of the agents leading to treatment failure (Hashem et al., 2022). Various side effects during the treatments pose multiple challenges to the patients; they would suffer from a functional loss in different behavioral and life domains and psychosocial distress (Concin et al., 2021). Hence, it presents an urgent need to explore new treatment alternatives for EC to improve patient outcomes and prognosis.

The specific pathogenesis of EC remains to be fully elucidated. Several physical, clinical, and genetic variables, including age, race, proximity to the metabolic syndrome, unopposed estrogen exposure, and genetic predispositions, are thought to have a role in the unique etiology of EC (Cai et al., 2019; Passarello et al., 2019). EC can be typically categorized into type-I and type-II due to their molecular and histopathology features, based on a classification system produced by Bokhman in 1983. Type-I accounts for most EC cases (70%–80%). In the endometrium, periodic hyperplasia is delicately controlled by programmed cell growth and death. The long-term effects of estrogen without progestin antagonism, which cause endometrial hyperplasia and atypical hyperplasia, followed by carcinogenesis, may cause type-I EC. Endometrial hyperplasia is a significant problem, and also the associated risk factors include hyperinsulinemia, obesity, high estradiol levels, and advanced age. Endometrial hyperplasia without atypical has a low (5%) risk of progression to endometrial cancer over 20 years. However, atypical glandular hyperplasia has a 27.5% risk of progression over 20 years and up to 43% of such patients (Hutt et al., 2019). Atypical hyperplasia, related to abnormal growth and proliferation of endometrial cells, is a precursor lesion for type-I EC (Armstrong et al., 2012; Braun et al., 2016; Urick and Bell, 2019), while its molecular basis is still unclear (Terzic et al., 2021). Apoptosis is a multistep programmed cell death process critical in clearing senescent and aberrant cells. Studies have demonstrated that inhibited cellular apoptosis is closely associated with the pathogenesis of EC. Dysfunction or inhibition of cellular apoptosis in the endometrium causes uncontrolled cell proliferation, aberration, and carcinogenesis (Fisher, 1994; Zhang et al., 2020)**.** Given this situation, regulating apoptosis of EC cells would be a promising target for developing effective anti-EC agents.

In recent years, natural products (NPs) have become a research hotspot in cancer treatment (Huang et al., 2019; Atanasov et al., 2021; Kim et al., 2021; Anjum et al., 2022; Liu et al., 2022; Huang et al., 2023; Yuan et al., 2023)**.** NPs refer to components, isolated metabolites, and extracts from natural plants and be of multiple bioactivities, such as regulating oxidative stress, inflammatory response, and cellular apoptosis. These agents also reveal therapeutic effects on various cancers (Shanmugam et al., 2016) with low toxicity and few side effects (Torquato et al., 2017). The detailed mechanisms underlying the anti-cancer properties of NPs need to be further explored to facilitate the development of NP-based anti-cancer agents. Both *in-vitro* and *in-vivo* studies demonstrate that many NPs could effectively suppress EC cells’ growth, proliferation, and differentiation via regulating apoptosis (Liu et al., 2012), indicating the apoptosis-regulatory properties of NPs would be a promising direction for further exploration.

Therefore, we have performed a comprehensive search in Google Scholar, PubMed, China National Knowledge Infrastructure (CNKI), Wanfang Database, and VIP database, from the inception to 31 December 2022, for studies regarding NPs for the treatment of EC via inducing apoptosis, and have reviewed the relevant pathways including mitochondria-dependent apoptotic pathway, endoplasmic reticulum stress (ERS) mediated apoptotic pathway, mitogen-activated protein kinase (MAPK) mediated apoptotic pathway, NF-κB mediated apoptotic pathway, PI3K/Akt mediated apoptotic pathway, p21-mediated apoptotic pathway and others. We hope our work could provide inspiration and valuable references for future studies.

## 2 Overview of apoptosis

Cellular apoptosis is a genetically-regulated programmed cell death process that plays an essential role in cellular metabolism (Hengartner, 2000). Inadequate apoptosis could cause pathological changes like carcinogenesis, autoimmune diseases, and diabetes (Nair et al., 2014). It was first reported by Kerr et al., in 1972, describing it as characteristic morphological changes and a series of enzyme-dependent biochemical processes (Kerr et al., 1972). Apoptosis can be divided into the exogenous death receptor and endogenous mitochondrial apoptosis pathways (Ricci and El-Deiry, 2007; Zhang et al., 2022).

The intrinsic pathway refers to apoptotic cascades triggered by intracellular signals, such as DNA damage, aberrant cell metabolism, calcium overload, chemotherapeutic drugs, radiation, high levels of reactive oxygen species, and detachment from the extracellular matrix (Chaudhry and Asselin, 2009). In a typical situation, there is a dynamic balance between the expression of pro-apoptotic protein and anti-apoptotic protein, which regulates physiological apoptosis. Decreased expression of anti-apoptotic protein BCL-2 family members or increased expression of pro-apoptotic proteins in response to the various stimulus signals described previously leads to an unbalance in the BCL/BAX ratio, which in turn initiates the endogenous pathway. The intrinsic pathway is mainly mediated by the B Cell lymphoma-2 (BCL-2) gene family (Ashkenazi, 2008; Nair et al., 2014). Interactions between the BCL-2 protein family determine mitochondrial outer membrane permeability (Green, 2022). The pro-apoptotic BCL-2 effectors, such as BAX, BAK, BIM, BID, and PUMA, promote apoptosis by causing mitochondrial outer membrane permeabilization (MOMP), whereas anti-apoptotic BCL-2 effectors inhibit this process, such as BCL-2, BCL-XL, BCL-W, BCL-2-A1 and MCL1 (Carneiro and El-Deiry, 2020; Wolf et al., 2022). When BAX/BAK is inserted into the mitochondrial membrane, cytochrome c (Cyt-c) is released into the cytosol from the outer mitochondrial membrane. Cytochrome c’s release is critical in cell apoptosis (Santucci et al., 2019). Cytosolic cytochrome c combines with apoptotic protease activating factor-1 (Apaf–1) and recruit pro-caspase-9 to form the apoptosome, a multiprotein complex (Li et al., 2017). Apoptosome is a multiprotein platform of caspase-9 activation to execute apoptosis (Malladi et al., 2009; Bratton and Salvesen, 2010; Dorstyn et al., 2018; Avrutsky and Troy, 2021). Once activated, caspase-9 could cleave and activate downstream pro-caspase-3 and -7 in the apoptosome, which in turn triggers the activation of further caspase-9 (Qin et al., 1999, 1; McComb et al., 2019, 7). If caspase-9 successfully processes some caspase-3 or caspase-7 in this situation, XIAP can bind to and suppress these active effector caspases (Bratton and Salvesen, 2010).

The extrinsic pathway is mainly triggered by extracellular stimuli (Jan and Chaudhry, 2019; Kashyap et al., 2021). The extracellular ligands such as tumor necrosis factor (TNF), Fas ligand (Fas-L), death receptor3 ligand (DR3L), and TNF-related apoptosis-inducing ligand (TRAIL) recognize and bind to their cognate death receptors (such as TNFR, Fas, DR3, DR4 or DR5) (Mandal et al., 2020). Procaspase-8 binds to the exposed DED of death receptor-related FADD through a pocket in its DED1 to form a death-inducing signaling complex (DISC) (Jiang M. et al., 2021) and subsequently activate pro-caspase-8. It can cleave and activate the downstream targeted molecules, including executor caspase-3 and caspase-7 and turn on the exogenous apoptotic cell death response (Jiang M. et al., 2021). Activated caspase-8 is a crucial protein of cross-talk signal way and could cleave Bid into tBid. Bid is generally thought to be inactive as an apoptosis inducer. tBid could induce mitochondrial outer membrane permeabilization (MOMP) in cells and induce the release of cytochrome c (CytC) and Smac/DIABLO from the mitochondria. Eventually, tBid can initiate the mitochondrial apoptosis pathway and makes significant in the endogenous apoptotic pathway by activating caspase-9 (Kantari and Walczak, 2011).

## 3 Endometrial carcinogenesis

Carcinogenesis in the endometrium is a complex and multistep process. The specific mechanisms remain elusive while several physical, pathological, and genetic factors are considered to be involved, such as age, race, concomitance with metabolic syndrome, unopposed estrogen exposure, and genetic predispositions (Cai et al., 2019; Passarello et al., 2019). Dysregulation of cellular apoptosis in the endometrium causes uncontrolled cell proliferation, aberration, and carcinogenesis (Fisher, 1994; Mirakhor Samani et al., 2018; Zhang et al., 2020).

EC can be typically categorized into type-I and type-II due to their molecular and histopathological features (Rodríguez-Palacios et al., 2022; Karia et al., 2023), based on a classification system produced by Bokhman in 1983. The type-I EC, endometrioid tumors, accounts for most EC cases (70%–80%). The type-I EC is derived from a precancerous condition called endometrial hyperplasia, whereas the type-II is hormone-independent pathogenesis without known precursor lesions (Huvila et al., 2013). Hyperplasia is a significant problem, and the associated risk factors include hyperinsulinemia, obesity, high estradiol levels, and increasing age (Singh et al., 2020). Endometrial hyperplasia without atypical has a low (5%) risk of progression to endometrial cancer over 20 years. However, atypical glandular hyperplasia has a 27.5% risk of progression over 20 years and up to 43% of such patients (Hutt et al., 2019). Atypical hyperplasia may further evolve into complex atypical hyperplasia (CAH). CAH is a precursor lesion for endometrioid-type endometrial cancer and is related to abnormal growth and proliferation of endometrial cells (Armstrong et al., 2012; Braun et al., 2016; Urick and Bell, 2019).

Clinical studies have found that the normal apoptotic mechanisms of many malignant cells are inhibited, preventing the body from early clearance of cells that may be at risk of cancer. Endometrial periodic hyperplasia is under delicate control by programmed cell growth and death. Apoptosis typically occurs between the human endometrium’s late secretory and menstrual stages (Otsuki, 2001). Compared to the proliferating phase, the expression of BCL-2 and the activation of caspase-3, -8, and -9 are higher in secretory to menstruating stages (Otsuki, 2001). It is reported that EC patients are resistant to apoptosis due to the unbalance of the anti- and pro-apoptotic molecules. Increasing evidence has suggested that anti-apoptotic mediators, such as BCL-2, Mcl-2, and IAP (Ai et al., 2006), are downregulated in EC patients, whereas the pro-apoptotic proteins, such as tumor necrosis factor-related apoptosis-inducing ligand (TRAIL), p53 (Kohlberger et al., 1996; Geisler et al., 1999; Edmondson et al., 2017) upregulated modulator.

Cellular apoptosis is crucial in endometrial hyperplasia, atypical hyperplasia, complex atypical hyperplasia, and eventually endometrial cancer. Given this situation, regulating apoptosis of EC cells would be a promising target for developing effective anti-EC agents and could provide a possible direction for developing anti-EC drugs. In almost all cases, detailed information of NPs and their potential effects with mechanisms on modulating apoptosis in EC is illustrated in Table 1, and the chemical structures of isolated metabolites are summarized in Table 2.

**TABLE 1:** Potential effects and mechanisms of natural products on modulating apoptosis in EC.

Potential pathways	Detailed mechanisms	Extracts/monomers (dose/concentration)	Cell/Animal model	Related targets	Refs
Mitochondria-dependent pathway	Up-regulating caspase-9, -3; Down-regulating bcl-2	Tian-Long compound (0.05%–0.5%)	Ishikawa cell	Caspase-9, -3, bcl-2	Li et al. (2009)
	Increasing p53 phosphorylation; Decreasing bcl-2	SDGE (0.025–12.50 μg/ml)	Ishikawa, ECC-1 cells	p53, bcl-2	Liu et al. (2012)
	Up-regulating bad, bak, bax; Up-regulating bcl-2, bcl-xL, caspase-9, -3, -8	SOE (50–150 μg/ml)	RL95-2 cell	bad, bak, bax, bcl-2, bcl-xL, caspase-9, -3, -8	Chang et al. (2014)
	Up-regulating caspase-3, bax; Down-regulating bcl-2	Zedoary Turmeric Oil (120–960 mg/L)	HEC-1B	caspase-3, bax, bcl-2	Li et al. (2021)
	Increasing DR5, bim, PUMA; Decreasing survivin	Flavokawain B (1.1–8.8 μM)	SK-LMS-1, ECC-1, T-HESC cells	DR5, bim, p53, survivin	Eskander et al. (2012)
	Incuding Ca2+ influx; Down-regulating bcl-2; Up-regulating bax, caspase-3, -9	Hyperin (0–500 μM)	RL95-2 cell	Bcl-2, bax, caspase-3, -9	Li et al. (2012)
	Decreasing bcl-2, bcl-xL; Increasing caspase-3, -9, PARP	Cucurbitacin D (0.5–4 μM)	Ishikawa, HHUA, HEC59	Bcl-2, bax, caspase-3, -9, PARP	Ishii et al. (2013)
	Decreasing bcl-2; Increasing p53, caspase-9, -3	Triptolide (10–320 nM)	HEC-1 B Cell	Bcl-2, p53, caspase-9, -3	Wang et al. (2014)
	Increasing ROS, caspase-9, -8, -3, cyto-c	α-terthienylmethanol (0–2 μM)	HEC-1A, Ishikawa cells	ROS, caspase-9, -8, -3, cyto-c	Lee et al. (2015)
	Decreasing bcl-2; Increasing caspase-3, PARP	Ginsenoside Rh2 (20, 40 μM)	Ishikawa, HEC-1A	Bcl-2, caspase-3, PARP	Kim et al. (2017b)
	Up-regulating caspase-3, bax; Down-regulating bcl-2; Increasing ROS, PARP, p-ERK1/2	Hinokitiol (1–50 μM)	Ishikawa, HEC-1A, KLE cells	Caspase-3, bax, bcl-2, PARP, ERK	Chen et al. (2021)
	Increasing cyto-c, caspase-3, -9, bax, bim; Decreasing bcl-xL XIAP, survivin	Curcusone C (0.1nM-100 μM)	HEC-1A, hESCs	Cyto-c, caspase-3, -9, bax, bim, bcl-xL XIAP, survivin	An et al. (2021)
ERS mediated stress	Activating GPR78; Increasing CHOP	Realgar quantum dots (0–30 μg/ml)	JEC cells	GPR78, CHOP	Li et al. (2009)
	Increasing Ca2+ influx, caspase-3, -7, CHOP, PARP	Cannabinoids (0.01–25 μM)	Ishikawa, Hec50co	Caspase-3, -7, CHOP, PARP	Fonseca et al. (2018)
	Increasing PERK, p-eIF2a, ATF4; Activating Hippo signaling pathway	Wogonoside (50μM, 80 mg/kg)	Ishikawa	PERK, p-eIF2a, ATF4, Hippo	Chen et al. (2019)
			BALB/c-nu mice		
	Up-regulating caspase-3, PARP, JNK, p38; Down-regulating ERK; Activating Akt	ProEGCG (20, 40, 60 μM)	AN3 CA, RL95–2 cells	Caspase-3, JNK, p38, ERK,Akt	Man et al. (2020)
MAPK mediated pathway	Activating ERK, JNK	Ellipticine (1–10 μM)	RL95-2 cell	ERK, JNK, caspase-7, -8, -9, -3, bid, XIAP, AIF, cyt-c	Kim et al. (2011)
	Up-regulating caspase-7, -8, -9. −3; Down-regulating Bid, XIAP				
	Increasing bax, ERK1/2; Decreasing bcl-2	Icaritin (0–10 μM)	HeC-1A	ERK, bax, bcl-2	Tong et al. (2011)
	Decreasing p-ERK; Increasing caspase-3	Annonacin (0.2–100 μg/ml)	ECCs cells	ERK, caspase-3	Chung et al. (2017)
	Up-regulating caspase-3, bax, bik; Down regulating bcl-2, ESR1	Hesperidin (5–50 μM)	ECC-1 cells	Caspase-3, bax, bik, blc-2, ESR1	Cincin et al. (2018)
	Up-regulating caspase-3, bax, p38, ERK, JNK, ROS; Down-regulating bcl-2, Akt	Emodin (1.25, 2.5, 5 μM)	KLE cells	Caspase-3, bax, bcl-2, p38, ERK, JNK, Akt	Jiang et al. (2019)
	Inhibiting ERK1/2 phosphorylation, c-Jun	Curcumin (10–80 μM)	Ishikawa	ERK, c-Jun	Zhang et al. (2019b)
NF-κB mediated pathway	Decreasing NF-κBp50; Up-regulating IκBα, caspase-3	Scutellaria baicalensis; Fritillaria cirrhosa (1.5–500 μg/ml)	EM-E6/E7/TERT, Ishikawa, HEC-1B Cells	NF-κBp50, IκBα, caspase-3	Kavandi et al. (2015)
	Inhibiting NF-κB; Down-regulating caspase-3	Curcumin (0–150 μM)	Ishikawa, HEC-1	NF-κB, caspase-3	Xu et al. (2018)
	Inhibiting VEGF/PI3K/Akt pathway	Panaxnotoginsengsaponins (50–200 μg/ml)	Ishikawa, HEC-1A cells	VEGF, Akt	Tan et al. (2016)
P13K/Akt/mTOR pathway	Decreasing p-AKT; Up-regulating caspase-3; Regulating Akt/mTOR pathway	Resveratrol (0.1, 100 μg); (25–200 μmol)	HeLa, Hec-1A, KLE, RL95-2, Ishikawa and EN1078D cells	p-AKT, caspase-3, mTOR, p38-AMPK	(Sexton et al., 2006) (Xu et al., 2020)
	Decreasing p-AKT, p-ERK1/2; Increasing caspase-3	Pseudolaric acid B (0.5–10 μmol/l)	Ishikawa cells	AKT, ERK, caspase-3	Wang et al. (2017)
	Modulating miR-106b/PTEN/AKT/mTOR pathway; Up-regulating caspase-3, bax; Down- regulating bcl-2	Shikonin ((10–20μM; 0.3–0.7 μg/ml)	Ishikawa, HEC-1A, KLE, RL95-2 cells	miR-106b, PTEN, AKT, mTOR, caspase-3, bax, bcl-2	Yin, 2016; Huang and Hu (2018)
	Increasing bax, Decreasing bcl-2, p-mTOR, p-Akt, p-P13K	Kaempferol (0–20 μM)	MFE-280	Bax, bcl-2, P13K, Akt, mTOR	Lei et al. (2019)
	Up-regulating bax; Down-regulating bcl-2, P13K, Akt, mTOR	Amygdalin (8–128 mg/L)	EECs, RL95-2, HEC-1B	Bax, bcl-2, P13K, Akt, mTOR	Ye et al. (2020)
	Decreasing P13K, Akt, mTOR Up-regulating bax, bak, bad, cyto-c, caspase-3, -9; Down-regulating bcl-xL	Asparanin A (6–18 μM)	Ishikawa cells	P13K, Akt, mTOR, bax, bak, bad, cyto-c, caspase-3, -9, bcl-xL	Zhang et al. (2020)
			Female BALB/c-nu mice		
	Up-regulating bax, caspase-3, -9, PARP, PETN; Down-regulating P13K, Akt	Osthole (25–200 μM)	EC-KLE, Ishikawa cells	P13K, Akt, PETN, bax, caspase-3, -9, PARP	Liang et al. (2021)
p21-mediated pathway	Regulating p53-independent pathway	Psammaplin A (1–10 μg/ml)	Ishikawa cells	p21^WAF1^, p53	Ahn et al. (2008)
	Down-regulating cyclin A, cyclin D3, bcl-2 and bcl-xL; Up-regulating p21^WAF1^, caspase-9	Bufalin (1 ng/ml)	Ishikawa, HHUS, HEC-1B, NHEEC cells	Cyclin A, cyclin D3, bcl-2 and bcl-xL, p21^WAF1^, caspase-9	Takai et al. (2008)
	Up-regulating p21; Down-regulating CDK4, MMP2, MMP9	Cinnamaldehyde (3.75, 7.5, 15 μg/ml)	Ishikawa cells	p21, CDK4, MMP2, MMP9	Dong and Li (2021)
Other	Not concluded	Rice bran fraction (100, 200, 300 μg/ml)	Sawano cell	Not concluded	Fan et al. (2000)
	Up-regulating BAG3, caspase-4, -5	PCAE (0–4 mg/ml)	Ishikawa cells	BAG3, caspase-4, -5	Tsai et al. (2015)
	Increasing ROS, bax; Decreasing bcl-2; Inhibiting pSTAT1, pSTAT2, pJAK1, pJAK2	Tanshinone l (0–40 μM)	HEC-1-A cells	Bax, bcl-2, STAT, JAK	Li et al. (2018)
	Up-regulating caspase-3, -7, PARP	Isoliquiritigenin (5–100 μM)	HEC-1-A, Ishikawa	Caspase-3, -7, PARP	Wu et al. (2016a)
	Inhibiting STAT3; Decreasing bcl-2, survivin	Silibinin (100, 150, 200 μM)	Ishikawa, RL-952	STAT3, bcl-2, survivin	Shi et al. (2019)
	Increasing miR-424 caspase-3, -9; Decreasing CPEB2	Osthole (50, 100, 200 μM)	Ishikawa, KLE	miR-424, CPEB2, caspase-3, -9	Lu et al. (2020)
	Increasing caspase-3	Gallic Acid (5–100 μg/ml)	Ishikawa cells	Caspase-3	Bulbul et al. (2021)
	Down-regulating XIAP, bcl-xL, pAKT via hnRNPA1	Esculetin (0–120 μM)	HEC-1B, Ishikawa cells	hnRNPA1, XIAP, bcl-xL, pAKT	Jiang et al. (2021b)
	Up-regulating caspase-3	Silymarin (6 μg/ml)	Ishikawa cells	caspase-3	Chen et al. (2019)

DR5, death receptor 5; PUMA, p53 Upregulated Modulator of Apoptosis; SDGE, steam distilled extract of ginger; SOE, siegesbeckia orientalis ethanol extract; CHOP, C/EBP, homologous protein; GPR78, G-protein coupled receptor 78; ERK, extracellular-signal-regulated kinase; JNK, c-Jun N-terminal kinase; ProEGCG, prodrug of (−)-epigallocatechin-3-gallate; AMPK, AMP-activated protein kinase; XIAP, X-linked inhibitor of apoptosis protein; AIF, apoptosis inducing factor; ESR1, estrogen receptor I; Cyto-c, cytochrome-c; β-HIVS, β-Hydroxyisovalerylshikonin; PARP, poly-ADP, ribose polymerase; BAG3, BCL-associated athanogene 3; VEGF, vascular endothelial growth factor; PCAE, pogostemon cablin aqueous extract.

**TABLE 2:** Detailed information and chemical structures of natural products.

Monomers	Origin	Systematic name	Chemical structures
Flavokawain B	Piper methysticum	(2E)-1-(2-Hydroxy-4,6-dimethoxyphenyl)-3-phenyl-2-propen-1-one	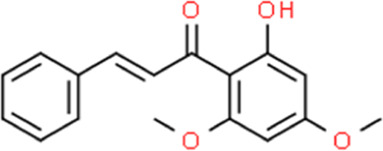
Hyperin	Rhododendron dauricum L.	2-(3,4-Dihydroxyphenyl)-5,7-dihydroxy-4-oxo-4H-chromen-3-yl β-D-galactopyranoside	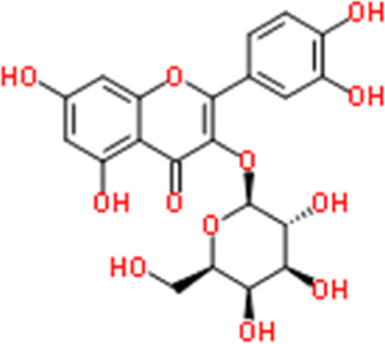
Cucurbitacin D	Pyrus communis subsp. communis	(2S,4R,9β,16α,23E)-2,16,20,25-Tetrahydroxy-9,10,14-trimethyl-4,9-cyclo-9,10-secocholesta-5,23-diene-1,11,22-trione	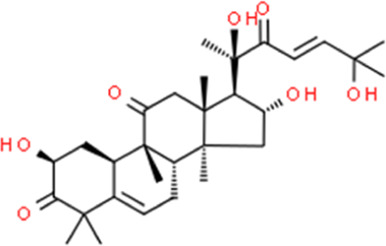
Triptolide	Tripterygium wilfordii Hook.f.	(3bS,4aS,5aS,6R,6aR,7aS,7bS,8aS,8bS)-6-Hydroxy-6a-isopropyl-8b-methyl-3b,4,4a,6,6a,7a,7b,8b,9,10-decahydrotrisoxireno [6,7:8a,9:4b,5]phenanthro [1,2-c]furan-1(3H)-one	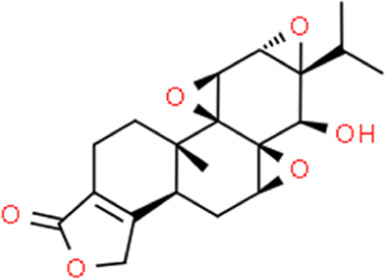
alpha-Terthienylmethanol	Eclipta prostrata (L.) L.	2,2':5′,2″-Terthiophen-5-ylmethanol	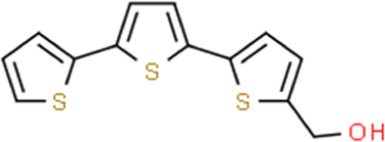
Ginsenoside Rh2	Panax ginseng C.A.Mey.	(3β,12β)-12,20-Dihydroxydammar-24-en-3-yl β-D-glucopyranoside	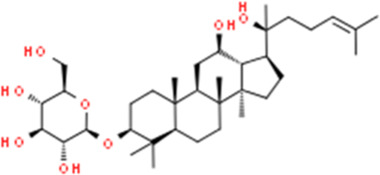
Hinokitiol	Chamaecyparis obtusa var. formosana (Hayata) Hayata	2-Hydroxy-4-isopropyl-2,4,6-cycloheptatrien-1-one	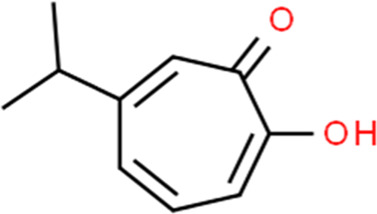
Curcusone C	Jatropha curcas L.	(2S,6aS)-2-Hydroxy-7-isopropenyl-2,5-dimethyl-10-methylene-2,3,6a,7,8,9,10,10a-octahydrobenzo [e]azulene-1,4-dione	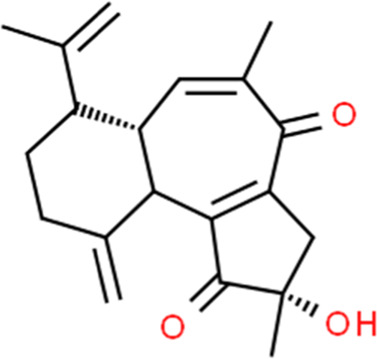
Wogonoside	Scutellaria baicalensis Georgi	5-Hydroxy-8-methoxy-4-oxo-2-phenyl-4H-chromen-7-yl β-D-glucopyranosiduronic acid	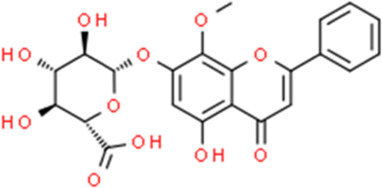
Ellipticine	*Ochrosia elliptica* Labill.	5,11-Dimethyl-6H-pyrido [4,3-b]carbazole	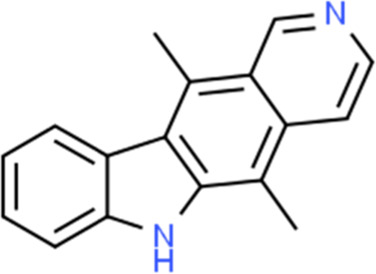
Icaritin	Epimedium brevicornu Maxim.	3,5,7-Trihydroxy-2-(4-methoxyphenyl)-8-(3-methyl-2-buten-1-yl)-4H-chromen-4-one	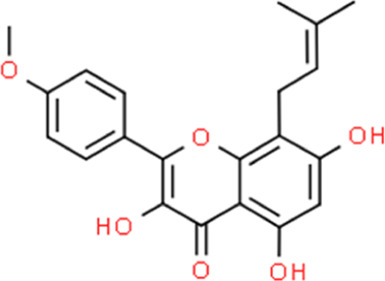
Annonacin	Annona muricata L.	(5S)-5-Methyl-3-[(2R,8R,13R)-2,8,13-trihydroxy-13-{(2R,5R)-5-[(1R)-1-hydroxytridecyl]tetrahydro-2-furanyl}tridecyl]-2(5H)-furanone	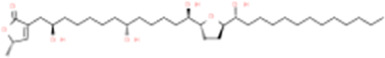
Hesperidin	Citrus × aurantium L.	(2S)-5-Hydroxy-2-(3-hydroxy-4-methoxyphenyl)-4-oxo-3,4-dihydro-2H-chromen-7-yl 6-O-(6-deoxy-α-L-mannopyranosyl)-β-D-glucopyranoside	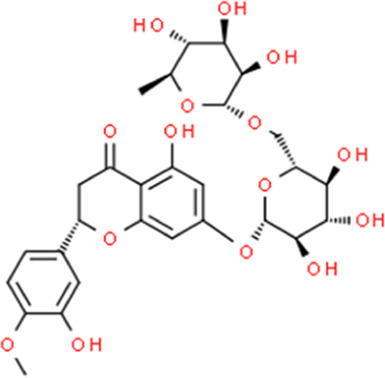
Emodin	Rheum palmatum L.	1,3,8-Trihydroxy-6-methyl-9,10-anthraquinone	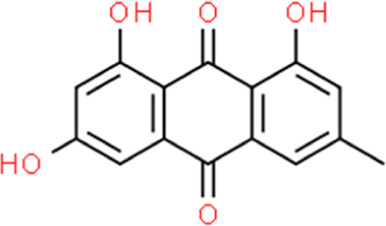
Curcumin	Curcuma longa L.	(1Z,6Z)-1,7-Bis(4-hydroxy-3-methoxyphenyl)-1,6-heptadiene-3,5-dione	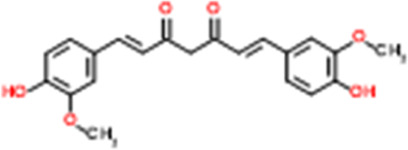
Scutellaria baicalensis	Scutellaria baicalensis Georgi	3-(9,9-Dimethyl-10(9H)-acridinyl)-N,N-dimethyl-1-propanamine	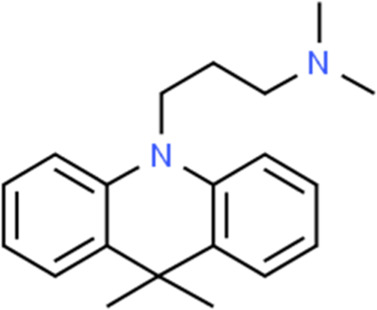
Resveratrol	Red wine	5-[(E)-2-(4-Hydroxyphenyl)vinyl]-1,3-benzenediol	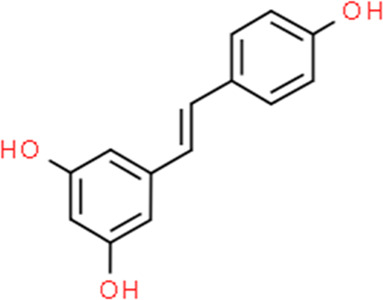
Pseudolaric acid B	Larix kaempferi (Lamb.) Carrière	(2E,4E)-5-[(1R,7S,8S,9R)-7-Acetoxy-4-(methoxycarbonyl)-9-methyl-11-oxo-10-oxatricyclo [6.3.2.01,7]tridec-3-en-9-yl]-2-methyl-2,4-pentadienoic acid	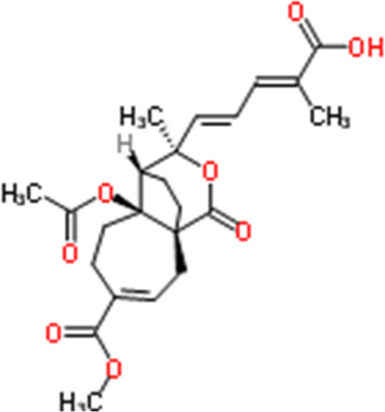
Shikonin	Lithospermum erythrorhizon Siebold & Zucc.	5,8-Dihydroxy-2-[(1R)-1-hydroxy-4-methyl-3-penten-1-yl]-1,4-naphthoquinone	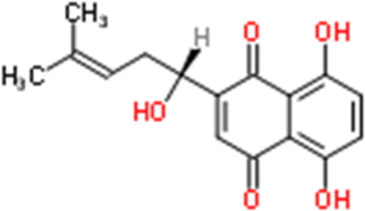
Kaempferol	Kaempferia galanga L.	3,5,7-Trihydroxy-2-(4-hydroxyphenyl)-4H-chromen-4-one	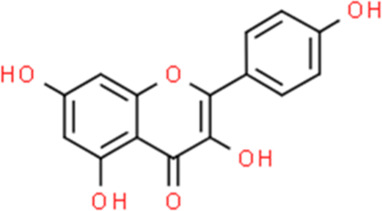
Amygdalin	Prunus amygdalus Batsch	(2R)-{[6-O-(β-D-Glucopyranosyl)-β-D-glucopyranosyl]oxy}(phenyl)acetonitrile	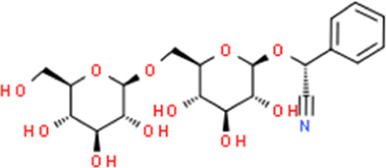
Asparanin A	Asparagus officinalis L.	(3β,5β,25S)-Spirostan-3-yl 2-O-β-D-glucopyranosyl-β-D-glucopyranoside	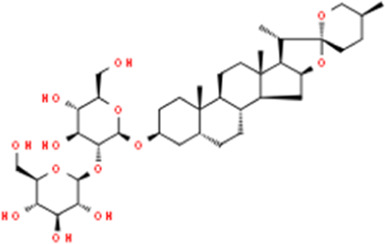
Osthole	Cnidium monnieri (L.) Cusson	7-Methoxy-8-(3-methyl-2-buten-1-yl)-2H-chromen-2-one	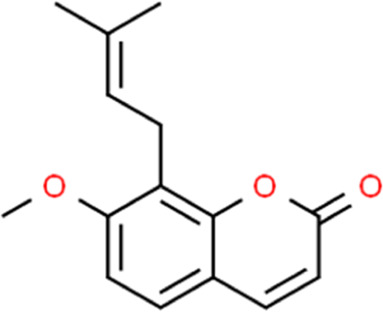
Isoliquiritigenin	Glycyrrhiza glabra L.	(2E)-1-(2,4-Dihydroxyphenyl)-3-(4-hydroxyphenyl)-2-propen-1-one	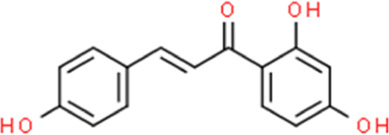
Esculetin	Fraxinus chinensis subsp. rhynchophylla (Hance) A.E.Murray	6,7-Dihydroxy-2H-chromen-2-one	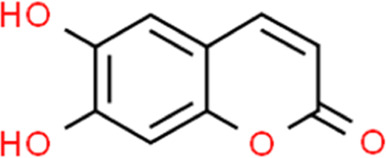
Silymarin	Silybum marianum (L.) Gaertn.	3,5,7-trihydroxy-2-[3-(4-hydroxy-3-methoxyphenyl)-2-(hydroxymethyl)-2,3-dihydro-1,4-benzodioxin-6-yl]-2,3-dihydrochromen-4-one	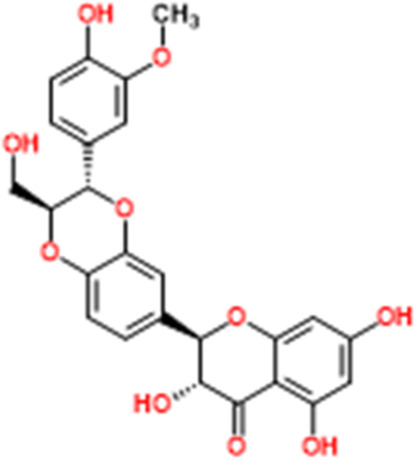

## 4 Effects and mechanisms of NPs on apoptosis in EC

### 4.1 Mitochondria-dependent apoptotic pathway

**FIGURE 1:**
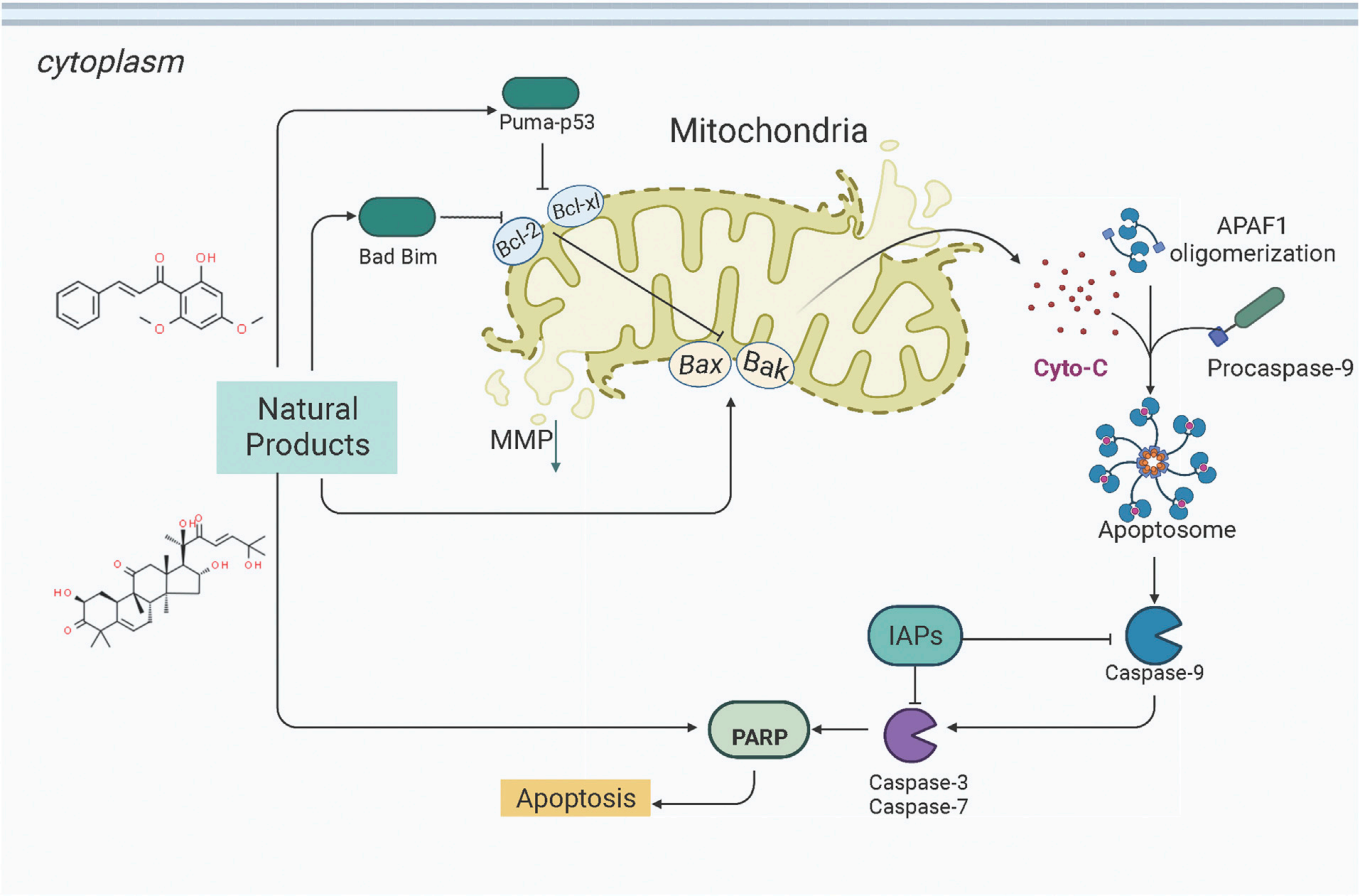
Natural products modulate apoptosis of ECCs through mitochondria-dependent pathway.

Mitochondria is the core organelle for energy synthesis and supply, thereby maintaining cellular function and managing cell life and death (Abate et al., 2020; Wang and Roh, 2020). Mitochondrial malfunction often triggers stress-mediated apoptosis. Since resistance to apoptosis is decisive for degenerative diseases and is a hallmark of cancer, the basis of cellular health is the correct functioning of mitochondria. Internal apoptotic signals, such as p53-PUMA or death receptor signal pathways, could alter the mitochondrial membrane permeability (MMP), releasing Cyto-c and other apoptosis-related factors into the cytosol to form the apoptosome. The apoptosome recruits and activates caspase-9, which in turn activates the effector caspases (caspase-3, -6, -7, *etc.*). Subsequently, the down-stream cascades by cleaving poly ADP-ribose polymerase (PARP) and actin substrates. Current studies demonstrate that some NPs effectively treat EC by modulating the mitochondria-dependent apoptotic pathway. All the relevant NPs that activate apoptosis via the mitochondria-dependent pathway are listed in Figure 1.

#### 4.1.1 Extracts from NPs

In early 2009, Li et al. studied the anti-EC effects of the Tian-Long compound (TL compound) *in vitro*. They found that TL compound (0.05%–0.5%) could significantly suppress the proliferation of Ishikawa cells by activating the mitochondrial-dependent apoptotic pathway. The potential mechanisms could be the upregulation of caspase-9 and caspase-3 and the downregulation of BCL-2 (Li et al., 2009). Liu et al. reported that a Steam Distilled Extract of Ginger (SDGE, 0.025–12.50 μg/ml) could induce apoptosis in Ishikawa and ECC-1 cells. The possible mechanisms are closely related to up-regulating p53 phosphorylation and down-regulating BCL-2 (Liu et al., 2012, 5). Later in 2014, Chang et al. investigated the pro-apoptotic effects of Siegesbeckia orientalis Ethanol Extract (SOE, 50–150 μg/ml) on Human Endometrial RL-95 Cancer Cells and found that SOE was of significant anti-proliferative and apoptotic effects in RL95-2 cells via activating both intrinsic and extrinsic signaling pathways. A study on its specific mechanisms revealed that SOE could upregulate the expression of Bad, Bak, and Bax, caspase-3, -9, and -8, whereas downregulate that of BCL-2 and BCL-xL (Chang et al., 2014). In 2021, Li et al. reported that Zedoary Turmeric Oil (120–960 mg/L) could significantly inhibit the proliferation of HEC-1-B cells and induce apoptosis by up-regulating the expressions of Bax and caspase-3 and down-regulating the expression of BCL-2 (Li et al., 2021).

#### 4.1.2 Monomers from NPs

In 2012, Zhou et al. reported that Flavokawain B (FKB, 1.1–8.8 μM) could significantly inhibit the growth of SK-LMS-1 and ECC-1 cell lines compared to non-malignant human endometrium fibroblast-like cells. The potential mechanisms might be associated with G2/M arrest and induction of mitochondrial-dependent apoptosis via upregulation of the pro-apoptotic proteins DR5, Puma, and Bim and downregulation of survivin, an inhibitor of apoptosis protein (IAP) (Eskander et al., 2012) and a promising therapeutic target as a new therapy for cancer treatment (Martínez-García et al., 2019). In 2012, Li et al. studied the anti-proliferative activity of Hyperin on RL952 cells. The results showed that Hyperin (0–200 μM) could suppress the viability of RL952 cells by inducing apoptosis, which would attribute to the regulation of Ca2+ influx, downregulation of BCL-2, and up-expression of bax, caspase-3,-8, and -9 (Li et al., 2012). In 2013, Cucurbitacin D (0.5–4 μM), extracted from Extrasynthese, proved the effect of induction of apoptosis via decreasing BCL-2, BCL-xL, and increasing caspase-3, caspase −9, PARP (Ishii et al., 2013). Triptolide (TP, 10–320 nM), a validated component purified from Tripterygium wilfordii Hook. f. showed to promote apoptosis via a p53-independent mitochondrial pathway. The possible mechanisms are closely related to the reactivation of the p53 to induce apoptosis via downregulation of the expression of BCL-2, and upregulation of caspase-9,-3 in HEC-1B Cells. In 2015, an *in vitro* study by Lee et al. suggested that α-terthienylmethanol (0–2 μM), isolated from Eclipta prostrata, and could induce apoptosis in HEC-1A and Ishikawa cells via increasing expression of Pro-caspase-3, 8, 9, and Cyto-c in a time-dependent manner and increasing ROS generation. The author also suggested that the apoptosis would be likely mediated by both the intrinsic and extrinsic pathways in ECCs (Lee et al., 2015). Later in 2017, Kim et al. found that the Ginsenoside Rh2 (20, 40 μM) could induce apoptosis in Ishikawa and HEC-1A cells via activation of caspase-3, PARP, and inhibition of BCL-2 (Kim J. H. et al., 2017). In 2021, Chen et al. observed that Hinokitiol (1–50 μM) could induce ROS-Mediated Apoptosis and p53-Driven Cell-Cycle Arrest in Endometrial Cancer Cell Lines (Ishikawa, HEC-1A, KLE) through up-regulating ROS,bax,caspase-3, PARP, p-ERK1/2, whereas down-regulating BCL-2 (Chen et al., 2021). In 2021, an *in vivo* study by Junxia et al. demonstrated that the Curcusone C (0.1 nM–100 μM) treatment caused significant anti-proliferative and apoptotic effects in Ishikawa and HEC-1A cells by inducing the release of Cytochrome c and increasing caspase-3,-9, Bax and bim, whereas decreasing X-linked inhibitor of apoptosis protein (XIAP), survivin, and BCL-xL (An et al., 2021).

### 4.2 Endoplasmic reticulum stress mediated pathway

**FIGURE 2:**
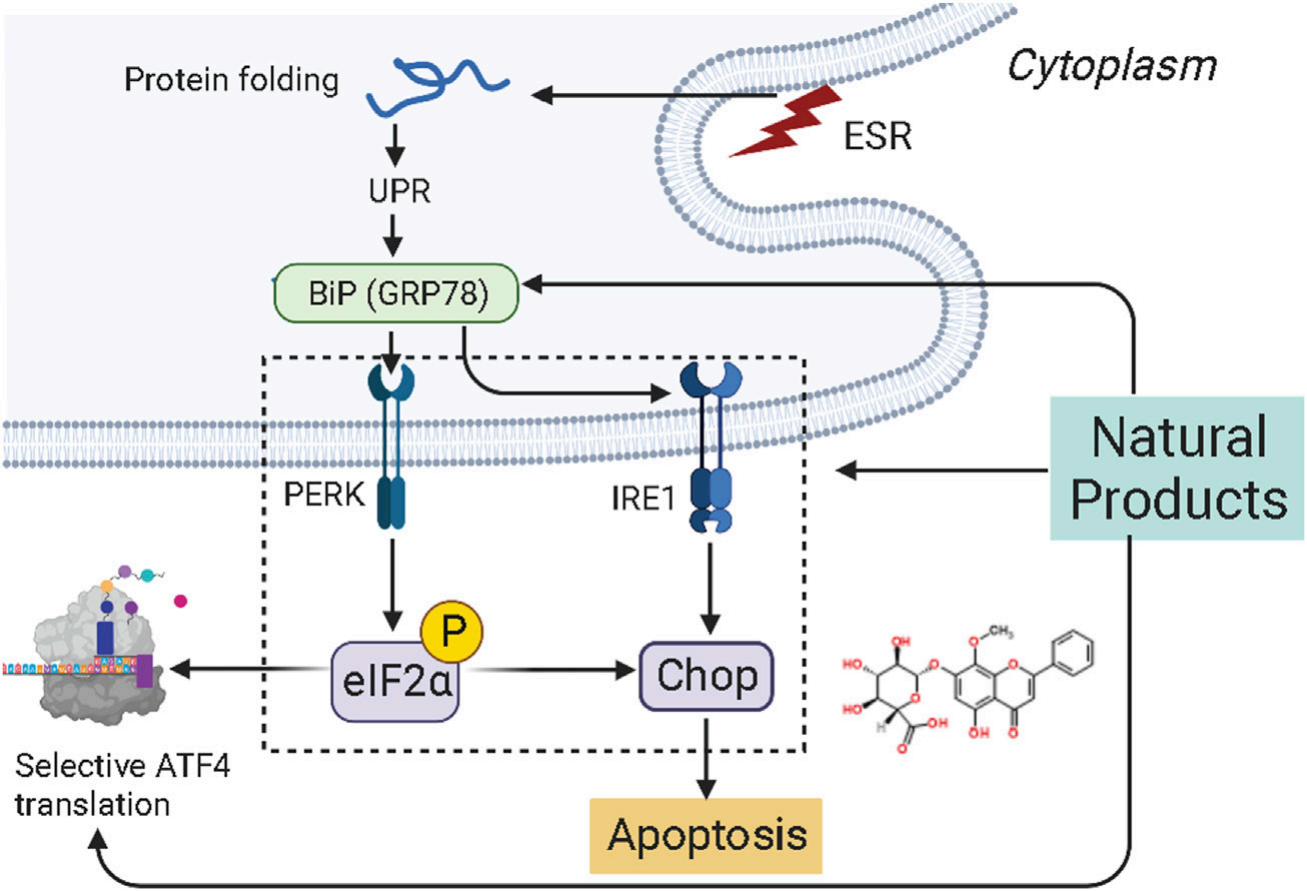
Natural products modulate apoptosis of ECCs through ERS-mediated pathway.

The endoplasmic reticulum (ER) is the central subcellular region for protein synthesis, folding, and transport. It also plays a vital role in intracellular Ca2+ homeostasis and various metabolic processes (Clarke et al., 2014; Wang et al., 2019). Cellular stress conditions can activate endoplasmic reticulum stress (ERS) to restore endoplasmic reticulum homeostasis and normal cellular function. In response to ER stress stimuli, such as the accumulation of unfolded/misfolded proteins in the ER above a critical threshold, the unfolded protein response (UPR) is initiated through three signaling cascades involving the protein kinase RNA-like ER kinase (PERK), inositol-requiring enzyme-1 (IRE1), and activating transcription factor-6 (ATF6) (Ron and Walter, 2007; Wang and Kaufman, 2016; Marciniak, 2019). However, if it fails, UPR triggers cell death (Wu F.-L. et al., 2016). ER stress and UPR have been shown to play critical roles in cancer pathogenesis, progression, and therapeutic response (Oakes et al., 2015). Increasing attention has been paid to ER stress’s essential role in endometrial carcinogenesis and the drug-resistance during chemotherapy. Several studies have demonstrated that NPs would be promising anti-cancer effects on EC via targeting ERS-mediated apoptosis. The potential effectiveness and mechanism of NPs on ERS-mediated apoptosis are summarized in Figure 2.

#### 4.2.1 Extracts from NPs

In 2015, Wang et al. found that Realgar quantum dots (RQDs, 0–80 μg/ml) can induce apoptosis *in vitro* by increasing the expression level of GRP78 (BIP) and GADD153 (CHOP). Their studies demonstrated that RQDs could activate ER stress and mitochondrial pathways (Wang et al., 2015). Later in 2018, Fonseca et al. reported that Cannabinoids (0.01–25 μM) could induce apoptosis *in vitro* by activating TRPV1 and increasing caspase-3,-7, Ca2+ influx, CHOP, and cleaved PARP (Fonseca et al., 2018).

#### 4.2.2 Monomers from NPs

In 2019, Chen et al. found that Wogonoside (50 μM, 80 mg/kg), a bioactive flavonoid component derived from Scutellaria baicalensis Georgi, can induce apoptosis and inhibit cell proliferation depending on the ER stress-Hippo signaling axis *in vitro* and *in vivo* (in Ishikawa and BALB/c-nu mice) via increasing the expression of protein kinase-like endoplasmic reticulum kinase (PERK), binding protein (Bip), p-eIF2a, and transcription factor 4 (TCF4) (Chen et al., 2019).

### 4.3 MAPK-mediated apoptotic pathway

**FIGURE 3:**
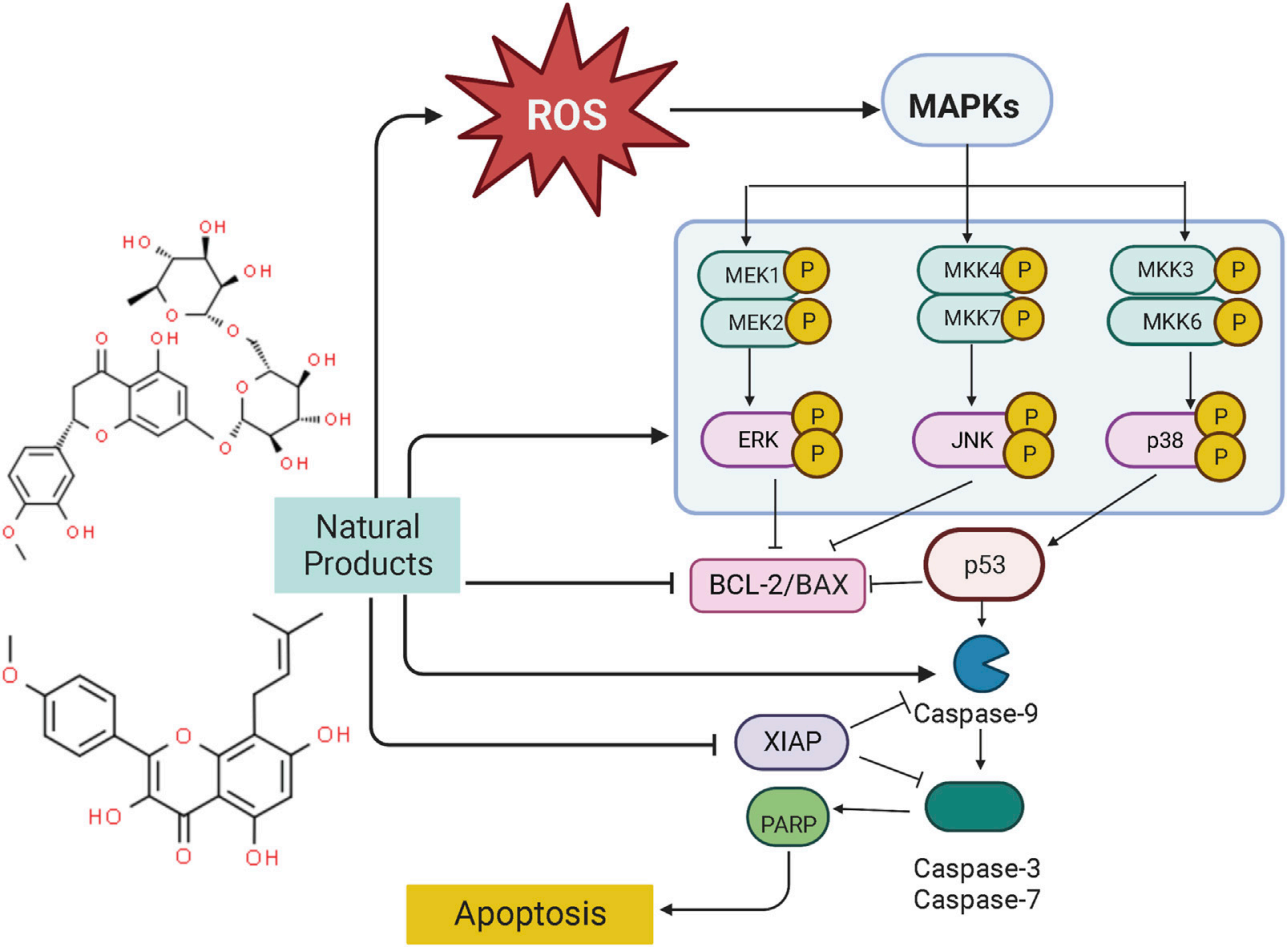
Natural products modulate apoptosis of ECCs through MAPK mediated pathway.

Mitogen-activated protein kinase (MAPK) pathway is an important signal transduction pathway in eukaryotic organisms. MAPK signaling pathways are involved in cell growth, migration, proliferation, differentiation, and apoptosis (Kim and Choi, 2010). Each MAPK signaling cascade consists of at least three layers of protein kinases: MAP3K, MAPKK, and MAPK. These cascades can be divided into extracellular signal-regulated kinase (ERK)1/2, c-Jun N-terminal kinase (JNK), P38 MAPK (P38), ERK3/4, and ERK7/8 (Chuderland and Seger, 2005; Dhillon et al., 2007). Among them, the JNK and p38 MAPK pathways are mainly related to cell stress and apoptosis, while ERK/MAPK signaling pathway is the most intensively studied MAPK signaling pathway, which is closely associated with cell proliferation and differentiation (Chuderland and Seger, 2005). However, the abnormal regulation of the MAPK signaling pathway plays a significant role in carcinogenesis. It is abundantly reported that NPs could be a promising way to treat EC to induce apoptosis through the MAPK pathway. The potential effectiveness and mechanism of NPs on MAPK-mediated apoptosis are summarized in Figure 3.

#### 4.3.1 Extracts from NPs

In another study by Man GCW et al., in 2020, the apoptotic effects of a prodrug of (−)-epigallocatechin-3-gallate (ProEGCG, 20, 40, 60 μM) showed a highly anti-proliferative activity on tumor cells in both EC xenografts cultured *in vivo* and RL95–2 and AN3 CA EC cells *in vitro* via promoting apoptosis, which was associated with activation of Akt, Up-regulating caspase-3, PARP, JNK, p38 whereas down-regulating ERK (Man et al., 2020).

#### 4.3.2 Monomers from NPs

Ellipticine (5,11-dimethyl-6H-pyrido [4,3-b]carbazole) is a bioactive component of Ochrosia elliptical, which has been demonstrated to be of pro-apoptotic effect on EC-RL95-2 cells (0.1–20 μM), and the potential mechanisms are related to the activation of ERK, JNA, as well as the increase of ROS generation. Ellipticine can also regulate the XIAP transcription and mediate the caspase cascade reaction to induce cellular apoptosis (Kim et al., 2011). In 2011, Tong et al. reported that Icaritin (0–10 μM), a compound from Epimedium Genus, possessed significant anti-proliferative and apoptosis-inducing activities in Hec1A cells, the potential mechanisms are correlated to increasing bax, ERK1/2 whereas decreasing BCL-2 (Tong et al., 2011). Another investigation in 2017 by Chung et al. studied the anti-proliferative effects of Annonacin (0.2–100 μg/ml) on both EC cell lines (ECC-1 and HEC-1A) and primary cells (EC6-ept and EC14-ept) and found that Annonacin has significant anti-proliferative activity via inhibition of ERK signaling pathway through down-regulating p-ERK whereas increasing caspase-3 (Chung et al., 2017). Hesperidin (Hsd) is the most active flavanone glycoside in citrus flavonoids. Studies in 2018 found that Hsd (5–50 μM) could downregulate MAPK, PI3K, STAT, and mTOR signal transduction pathways for regulating apoptotic and autophagic responses. The underlying mechanism may be related to up-regulating caspase-3, bax, and bik, whereas downregulate BCL-2 and ESR1 (Cincin et al., 2018). In 2018, Jiang et al. found that Emodin (1.25, 2.5, 5 μM), a significant component of rhubarb, can induce apoptosis *in vivo* (Xenograft Tumor Models) and *in vitro* in a time- and dose-dependent manner via inhibiting the PI3K/Akt pathways while activating MAPK signaling, and after Emodin treatment, caspase-3, bax, p38, ERK, JNK, and ROS were significantly upregulated whereas BCL-2 and Akt were downregulated (Jiang et al., 2019). Curcumin (10–80 μM), reported to have antioxidant, anti-inflammatory, liver protection, analgesia and antiarthritis, lipid modification, immune regulation, and anti-diabetic properties, could induce apoptosis via Inhibiting the Phosphorylation of ERK/c-Jun pathway via reducing mRNA expression of ERK2 and JUN genes (Zhang Z. et al., 2019).

### 4.4 NF-κB mediated apoptotic pathway

**FIGURE 4:**
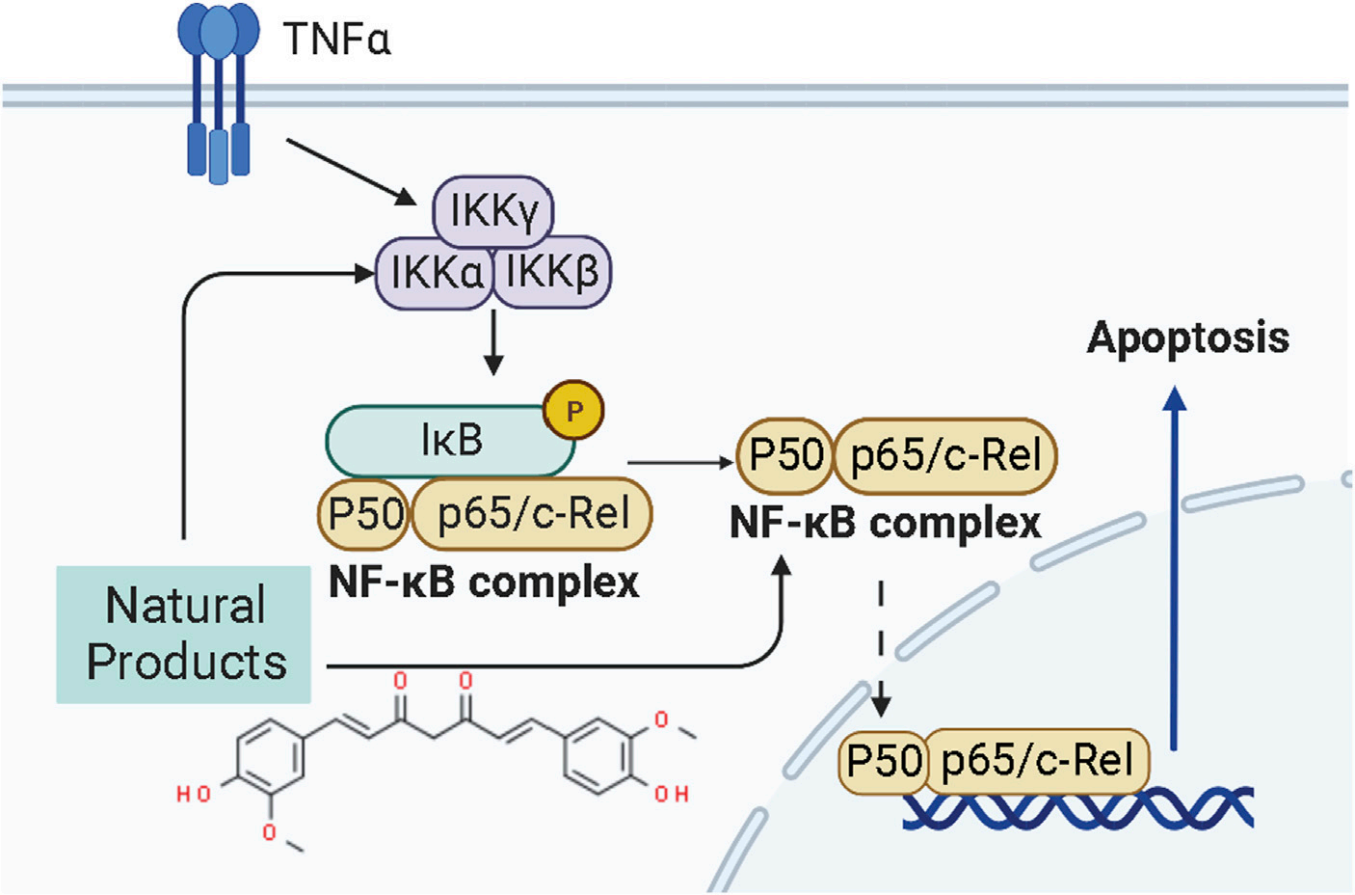
Natural products modulate apoptosis of ECCs through NF-κB mediated pathway.

NF-κB is a transcription factor that usually exists as a dimer. p65/relA and p50 are the most common dimeric forms of NF-κB, and its dimers have two states: inactivation and activation. In the “resting” state of cell c, NF-κB is inactive and binds to the inhibitor IκBα on the cell membrane, preventing it from entering the nucleus to activate genes. When external signals stimulate the cell, IκBα is degraded, NF-κB is released, and its nuclear localization sequence (NLS) is exposed. NF-κB rapidly enters the nucleus from the cell membrane and binds to specific sequences on nuclear DNA to initiate or enhance transcription of related genes, which can control protein transcription and participate in physiological processes such as cell proliferation and apoptosis, stress response, and cytokine release. Recently, the NF-κB pathway has been considered a promising therapeutic target for EC therapy. Studies have shown NPs can induce apoptosis in ECCs and prevent endometrial hyperplasia. The potential mechanisms of NPs on NF-κB mediated apoptosis are summarized in Figure 4.

In 2015, it was reported by Kavandi et al. found that the anti-proliferative properties of the herbs Scutellaria baicalensis (SB) and Fritillaria cirrhosa (FC,1.5–500 μg/ml) on EM-E6/E7/TERT, Ishikawa, and HEC-1B Cells closely related to NF-κB pathway via regulation of decreasing NF-κB p50 whereas up-regulating IκBα and caspase-3 (Kavandi et al., 2015). Afterward, Xu et al., in 2018 recorded that Curcumin (0–150 μM) extracted from the rhizome of the plant Curcuma longa could induce apoptosis through negative regulation of the NF-κB pathway *in vitro in vivo*, and the molecular mechanisms might be related to inhibiting NF-κB and down-regulating caspase-3 (Xu et al., 2018).

### 4.5 PI3K/AKT/mTOR pathway

**FIGURE 5:**
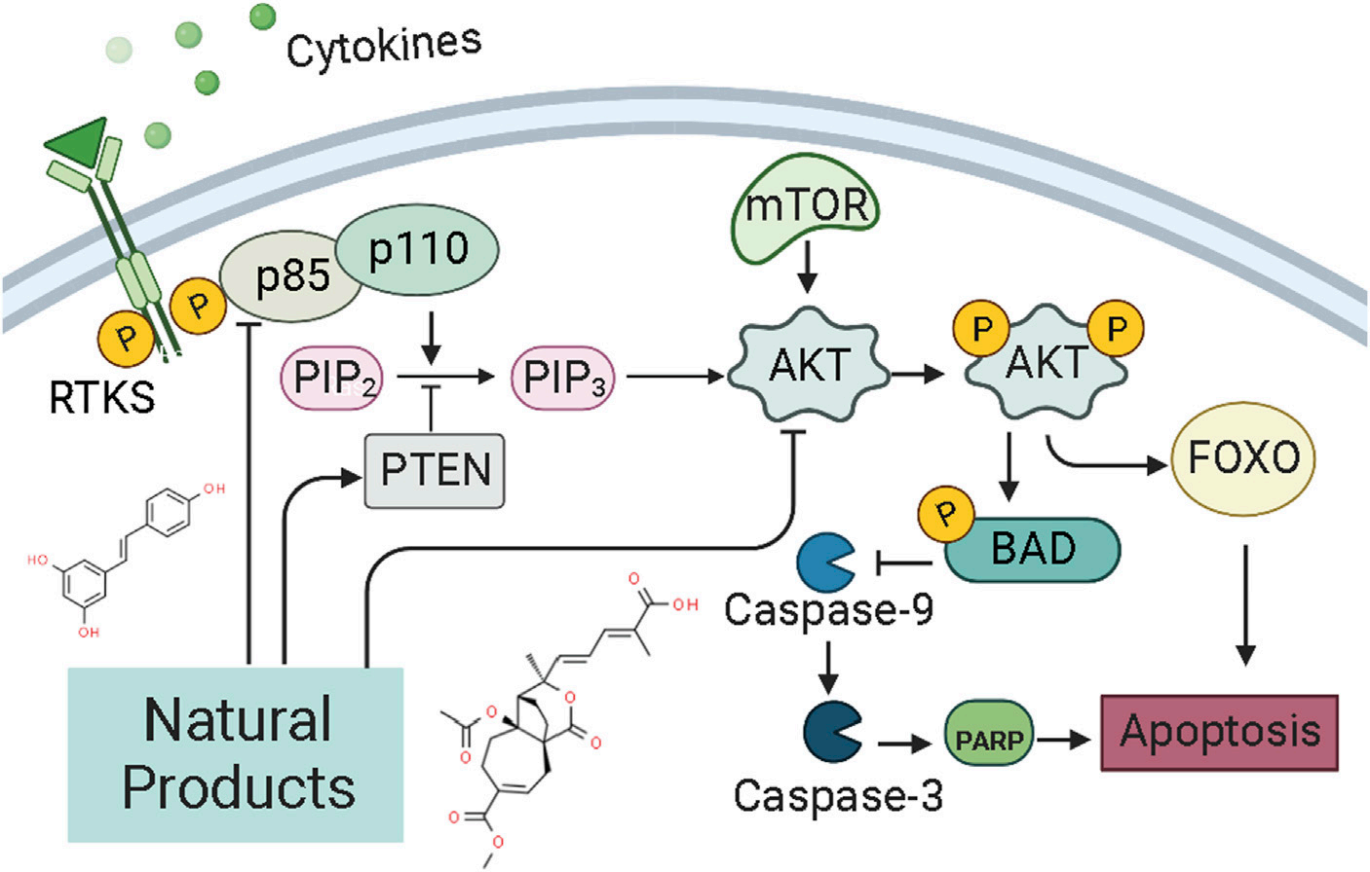
Natural products modulate apoptosis of ECCs through PI3K/AKT/mTOR pathway.

PI3K, or phosphatidylinositol 3-kinase, is a family of lipid kinases that control different processes in mammalian cells, including cell proliferation, survival, differentiation, activation of effector functions, and metabolism (Ali et al., 2015). The PI3K family consists of three classes of PI3Ks (I-III) (Narita et al., 2002). Class I can be further divided into class IA and class IB enzymes, and Class IA PI3K enzymes include a catalytic (p110) and a regulatory subunit (p85 or p101) (Hennessy et al., 2005; Sujobert and Sujobert, 2005; Fruman, 2008, 110; Piddock et al., 2017). Akt, also known as protein kinase B (PKB), is a serine/threonine-specific protein kinase (Yip, 2015). Signaling pathways determined by PI3K, AKT, and the mammalian target of rapamycin (mTOR) are critical for many features of cancer, such as cell growth, survival, metabolism, apoptosis, and angiogenesis (Ediriweera et al., 2019; Fattahi et al., 2020; Miricescu et al., 2020; Mirza-Aghazadeh-Attari et al., 2020). The PI3K/Akt/mTOR intracellular signaling cascade begins with activating RTKs and cytokine receptors, which generate phosphorylated tyrosine residues that provide anchor sites for recruiting PI3K to membrane translocation. Class IA PI3Ks can be activated by receptor tyrosine kinases (RTKs), G protein-coupled receptors (GPCRs) located on the cell surface membrane (Darici et al., 2020). Upon activation, The P110 catalytic subunit of PI3Ks could convert phosphorylate PI(4,5)P2 to PI(3,4,5)P3 (Denley et al., 2009), a second messenger. And then, PIP3 induces the activation of phosphoinositide-dependent kinase-1 (PDK1) and downstream targets of AKT (Pothongsrisit and Pongrakhananon, 2021). The levels of PI(3,4,5)P3 and PI(4,5)P2 could be regulated by PTEN (Chalhoub and Baker, 2009; Blanco et al., 2020). The PI3K/AKT/mTOR signaling pathway is the essential cell signaling pathway in animals, involved in regulating physiological processes such as cell growth, survival, proliferation, metabolism, and apoptosis. Alterations in the PI3K/AKT/mTOR pathway are now thought to be strongly associated with the carcinogenesis and progression of endometrial cancer (Slomovitz and Coleman, 2012; Chen et al., 2014). The pathway most frequently damaged in endometrial cancer is the PI3K/AKT/mTOR pathway (Crosbie et al., 2022). In recent years, it has been abundantly reported that NPs could exert pro-apoptosis via the PI3K/AKT/mTOR pathway (Mirza-Aghazadeh-Attari et al., 2020). The potential mechanisms of NPs on PI3K/AKT/mTOR mediated apoptosis are summarized in Figure 5.

#### 4.5.1 Extracts from NPs

In 2016, Tan et al. reported that the intervention of Panaxnotoginsengsaponins (PNS, 50–200 μg/ml) could induce apoptosis in Ishikawa and HEC-1A cells via inhibiting the expression of VEGF, which may be related to inhibiting PI3K/AKT/mTOR signaling pathway (Tan et al., 2016).

#### 4.5.2 Monomers from NPs

Resveratrol (3, 4, 5-trihydroxy-trans-stilbène), a natural phytoalexin present in grape skins, has considerable anti-proliferation effects and can induce apoptotic cell death in various types of cancers cell *in vitro*. In 2006, Émilie Sexton et al. reported that high-dose of resveratrol (0,10, and 100 μM) could inhibit cell growth and trigger apoptotic cell death *in vitro* via decreasing p-Akt, whereas up-regulating caspase-3 (Sexton et al., 2006). Another study in 2020 by Xu et al. also suggested that the anti-proliferative and pro-apoptotic effect of resveratrol might attribute to the regulation of the Akt/mTOR signaling pathway (Xu et al., 2020). Pseudolaric acid B (PAB) is the major bioactive component of Pseudolarix kaempferi Gorden. Studies in 2017 by Wang et al. have found that PAB (0.5–10 μmol/l) could inhibit Ishikawa cell proliferation and induces apoptosis *in vitro*. Its related molecular mechanisms may involve Akt-GSK-3β and ERK1/2 signaling pathways via decreasing p-Akt and p-ERK1/2 whereas increasing caspase-3 and p-GSK3β (Wang et al., 2017). Shikonin, an active biological component derived from the roots of the herb Lithospermu erythrorhizon, has considerable antitumor effects, including antioxidation, anti-inflammation, and anti-apoptosis. Studies reported in 2016 by Yin et al. and in 2017 by Huang et al. suggested that Shikonin could promote apoptosis *in vitro* by modulating the miR-106b/PTEN/Akt/mTOR pathway (Yin, 2016; Huang and Hu, 2018). In 2019, a study by Xia et al. suggested that Kaempferol (0–20 μM) can promote apoptosis via increasing bax whereas decreasing p-PI3K p-mTOR, p-Akt, and BCL-2 (Lei et al., 2019). Besides, from the results of Ye et al., Amygdalin (8–128 mg/L) could also induce apoptosis *in vitro* via regulation of the proteins related to the PI3K-Akt signal (Ye et al., 2020). Another study in 2020 by Zhang et al. first reported that Asparanin A (AA, 6–18 μM) could promote apoptosis *in vitro* and *in vivo* by activating the mitochondrial pathway and inhibiting PI3K/Akt signaling pathway (Zhang et al., 2020). Recently, a study by Liang et al., in 2021 showed that Osthole (25–200 μM) could suppress the growth *in vitro* and *in vivo*, which was associated with up-regulating bax, caspase-3, -9, PARP, PETN, whereas down-regulating PI3K and Akt (Liang et al., 2021).

### 4.6 P21-mediated pathway

**FIGURE 6:**
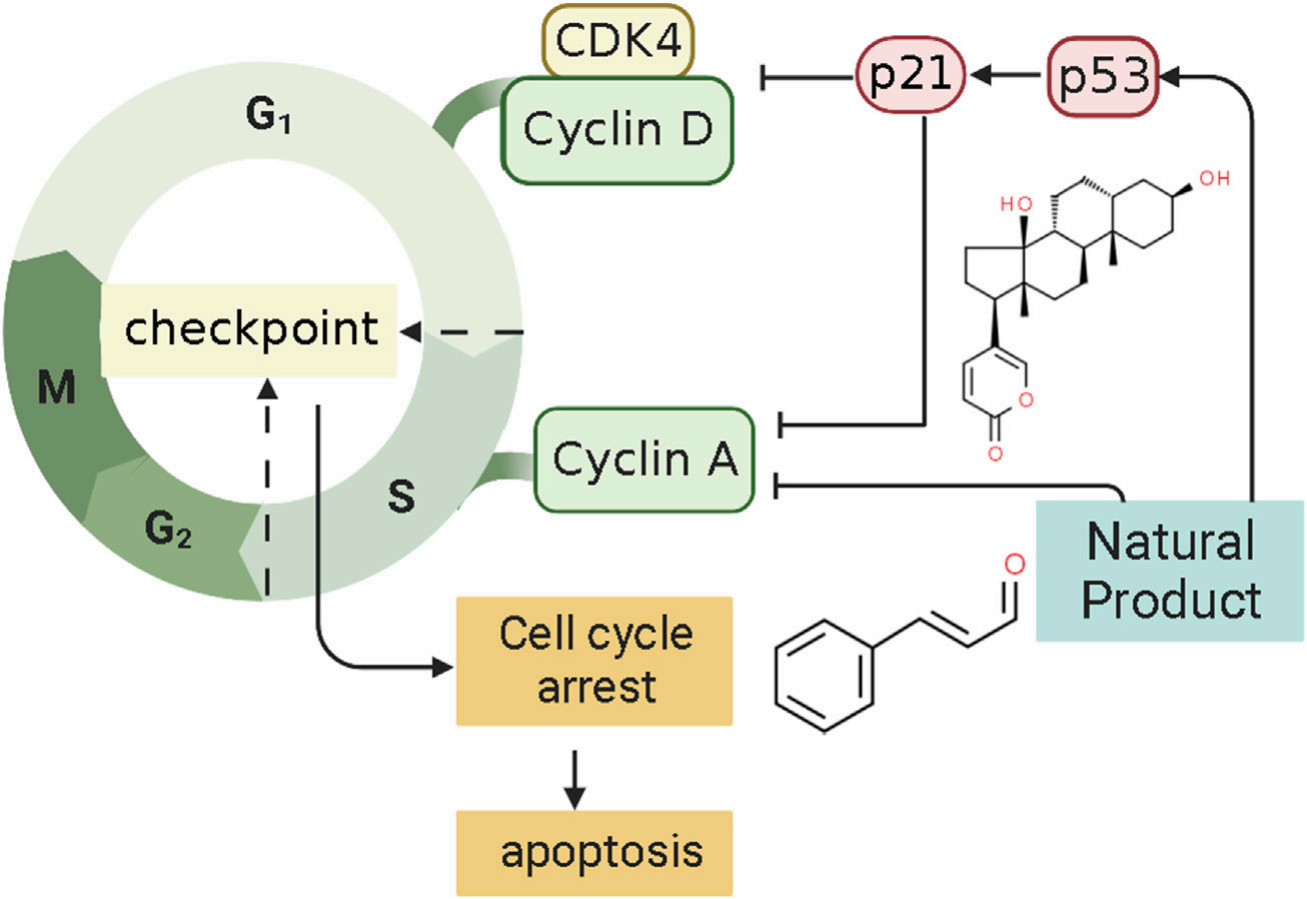
Natural products modulate apoptosis of ECCs through P21-mediated pathway.

P21, also called P21^WAF1^/CIP1 or P21/CDKN 1a, is a small protein with 165 amino acids related to cell cycle progression (Karimian et al., 2016). In 1993, a finding found that P21, or wild-type p53-activated fragment 1 (WAF1), is directly regulated by P53 and can suppress tumor cell growth in culture (El-Deiry et al., 1993). However, P21 is a downstream mediator of the P53 transcription factor and can interact directly with P53 (Kim E. M. et al., 2017). Thus, P21 may be an essential p53 growth suppression pathway component. Some findings indicate that the p53/p21 complex regulates cell apoptosis by targeting Bcl-2 proteins (Kim et al., 2022). P21 protein, a cyclin-dependent kinase inhibitor (CKI), can bind to and inhibit the activity of CDK1, CDK2, and CDK4/6 enzyme complexes (Marchetti et al., 1996), thereby acting as a cell cycle regulator at the G1 and S phases (Kikuchi et al., 2022). When DNA is damaged, the increased expression of p53 could activate the transcription of gene p21 by binding to its response element within its promoter. P21^WAF1^ can decrease kinase activity and may be a key regulator of G0/G1 accumulation and G1 cell cycle arrest. Consequently, cell apoptosis was induced by p21. Several studies have shown that NPs can regulate the cell cycle of ECCs by mediating P21, thereby promoting the induction of apoptosis in EC. The potential effectiveness and mechanism of NPs on P21-mediated apoptosis are summarized in Figure 6.

In 2008, after the human endometrial Ishikawa cancer cell line was prepared, Mee et al. investigated the effect of the Psammaplin A (0.1–10 μg/ml), a natural histone deacetylase inhibitor, induces on the Ishikawa cells. They found that Psammaplin A (5 μg/ml) can notably inhibit the proliferation and induced cell cycle arrest or apoptosis *in vitro*. The molecular mechanisms might be related to the increased expression of p21WAF1 through a p53-independent pathway (Ahn et al., 2008). In the same year, Takai et al. first demonstrated that Bufalin (1 ng/ml) could inhibit proliferation and induce apoptosis *in vitro*. The mechanism may be related to an increase in cleaved caspase-9 expression caused by up-regulating the levels of p21WAF1 protein and down-regulating cyclin A, cyclin D3, BCL-2, and BCL-xL (Takai et al., 2008). Recently, Dong et al. investigated the effect of Cinnamaldehyde (3.75, 7.5, 15 μg/ml) on Ishikawa cells. The results showed that Cinnamaldehyde has notable pro-apoptotic effects via up-regulating p21WAF1, whereas down-regulating CDK4, MMP2, and MMP9 (Dong and Li, 2021).

### 4.7 Other reported pathways

In addition to the apoptotic pathways mentioned above, there are NPs reported to exert pro-apoptotic effects on EC through other mechanisms.

#### 4.7.1 Extracts from NPs

In 2000, the Rice bran fraction (100, 200, 300 μg/ml) was reported to be a lipoprotein fraction that could induce apoptosis of the Sawano cells (Fan et al., 2000). In 2015, Tsai et al. studied the influence of Pogostemon cablin Aqueous Extract (PCAE, 0–4 mg/ml) on the induction of apoptosis. The results showed that PCAE induced apparent apoptosis in Ishikawa cells. In addition, further investigation revealed that the mechanism might be related to up-regulating BAG3,caspase-4, and caspase-5 (Tsai et al., 2015). In 2018, it was also reported that Tanshinone l (0–40 μM) could induce apoptosis and can increase ROS, bax. In contrast, downregulate BCL-2 and inhibit the phosphorylation of pSTAT1, pSTAT-2, pJAK1, and pJAk, inhibiting JAK/STAT pathway signal pathway and mitochondrial-mediated apoptosis in HEC-1-A cells (Li et al., 2018)**.**

#### 4.7.2 Monomers from NPs

In 2016, Wu et al. studied the inhibitory effect of Isoliquiritigenin (ISL, 5–100 μM), a licorice flavonoid, which was shown to could induce apoptosis and cell growth inhibition *in vitro* and *in vivo* via up-regulating caspase-3, caspase −7 and PARP (Wu C.-H. et al., 2016). Later in 2019, Shi et al. found that the Silibinin (SB, 100, 150, 200 μM), extracted from milk thistle seeds, can significantly inhibit the proliferation and promote apoptosis in a dose- and time-dependent manner via blocking pathways of STAT3 activation and SREBP1-mediated lipid accumulation, which is closely related to inhibiting STAT3, whereas decreasing BCL-2 and survivin (Shi et al., 2019). Lu et al., in 2019 recorded that Osthole (50, 100, 200 μM) could induce apoptosis in the Ishikawa and KLE cells, and after Osthole treatment,caspase-3, -9,miR-424 were significantly upregulated, and the CPEB2 were downregulated (Lu et al., 2020). A report in 2021 by Bulbul et al. studied the effect of Gallic Acid (3,4,5-tri hydroxybenzoic acid; GA; 5–100 μg/ml) and found it could induce apoptosis in Ishikawa cells by mitochondrial pathway via up-regulating caspase-3 (Bulbul et al., 2021). In 2021, Jiang reported that Esculetin (0–120 μM) could result in apoptosis and an arrest in proliferation in the HEC-1B, Ishikawa cells, and can target hnRNPA1, thereby downregulate the expression level BCL-XL, XIAP, and pAkt protein (Jiang R. et al., 2021). Recently, Hua et al. suggested that Silymarin (6 μg/ml) could induce apoptosis in Ishikawa cells via up-regulating caspase-3 (Chen et al., 2019).

## 5 Perspectives and conclusion

NPs are a wide range of bioactive components isolated from natural organisms, including plants, animals, insects, marine organisms (Manoharan and Perumal, 2022), and microorganisms. NPs are attractive sources for developing new medicinal and therapeutic agents (Thomford et al., 2018; Thompson and Lutsiv, 2023; Rao et al., 2019). For a long time, NPs have been regarded as a rich source of the active ingredients in new drugs. Moreover, the structural complexity and functional diversity of NPs are irreplaceable advantages compared to chemical drugs. These bioactive elements exert remarkable therapeutic effects on various diseases. NPs possess anti-cancer, anti-inflammatory, antioxidant, anti-bacterial, analgesic, anti-diabetic, and enzyme-inhibitory activities (Hassan et al., 2022). In recent years, the anti-cancer effects of NPs have drawn increasing attention (Hassan et al., 2022; Islam, 2022; Nuzzo et al., 2022), and we focus on their apoptosis-regulatory effect. Existing studies suggest that NPs can promote EC cell apoptosis through multiple pathways and thus exert anti-EC effects. Although existing research has reached a depth, some issues have not been well addressed and cannot be ignored to advance the development of NP-based anti-EC drugs.

First, the material basis of NPs for preventing and treating diseases is their active ingredients. Limited sources or meager amounts of bioactive molecules raw material is considered one of the most important obstacles to developing NPs into drugs. Many unexplored natural resources, especially uncultured marine organisms, will expand the sources of NPs because they can provide complex molecules with biologically active pharmacophores (Bilal and Iqbal, 2020). To better develop NPs, two different aspects may be involved: isolating additional structures directly from NPs, and modifying or improving these structures by chemical or biochemical methods (Li and Lou, 2018). These pathways may be helpful to facilitate the production of candidate molecules with lower costs, better efficacy, and less toxic side effects. Furthermore, the difficulty of extracting bioactive molecules is considered one of the significant obstacles to developing NPs into chemotherapeutic agents. Conventional extraction techniques frequently include preparatory fractionation of the parent material or crude extract, which limits their practical adoption on a large scale. Traditional extraction techniques also have other drawbacks, such as long extraction times, solvent purity issues, excessive solvent consumption and evaporation, shortened extraction yields, and thermal degradation of thermally degraded compounds. These limitations limit the development of NPs. Numerous modern extraction methods have been created and used, taking into account the structural and compositional characteristics of target sources, such as enzyme-assisted extraction (EAE), supercritical-fluid extraction (SFE), and microwave-assisted extraction (MAE), *etc.* (Bilal and Iqbal, 2020). Second, most studies have only focused on a single certain NP, and the combination of multiple NPs may help improve the efficacy and further explore the role of these NPs in the overall regulation of apoptosis, as well as their drug interrelationships. Third, the above studies were almost carried out via *in vitro* and *in vivo* approaches. Not all papers conducted *in vivo* experiments, so further investigation is suggested. Besides, the experimental data above almost explored a single pathway targeting the pro-apoptotic effects of NPs, and only a few NPs targeting cross-talk are available in studies. This may lead to the failure of drugs if this mechanism is interrupted or altered due to various cancer-related phenomena. This may also be a limitation and cause drug resistance to cancer. Clinical trials are also necessary to demonstrate whether the *in vitro* and *in vivo* animal data are reproduced in humans and to allow the application of NPs in cancer prevention and treatment. Most articles have analyzed their mechanism of action at the cellular and/or molecular level (Ekiert and Szopa, 2022). Fourth, NPs that are well tolerated and have less toxicity will help patients to achieve better treatment outcomes and improve their quality of life. The toxicity and pharmacokinetic selectivity of NPs should be further explored to validate their safety, which is a key step in the development of new drugs and can provide a strong basis for their translation to the clinic. Despite efforts to improve the therapeutic outcome for EC over the past decades, chemoresistance and side effects remain significant problems. The following clinical research stage must include a rational combination of agents that activate apoptotic signaling pathways and block pro-survival mechanisms while minimizing off-target toxicities. Furthermore, NPs that can treat various symptoms related to chemotherapeutics, such as nausea and vomiting, should be investigated. Exploration of the combination NPs with classical chemotherapeutic agents may be a possible way to enhance the susceptibility of cancer cells. Moreover, a study (Li et al., 2022) suggests that acupoint stimulation involves synergy with chemotherapy and can alleviate chemotherapeutic agents side effects.

NPs with anti-EC effects were classified and systematically organized by their inducing-apoptosis mechanisms and the sources in the review. The cell line, animal model, dose, efficacy, and mechanism of the NPs in each paper were covered clearly. The main related signal pathways are the mitochondrial-dependent apoptotic pathway, endoplasmic reticulum stress (ERS) mediated apoptotic pathway, mitogen-activated protein kinase (MAPK) mediated apoptotic pathway, NF-κB mediated apoptotic pathways, PI3K-Akt mediated apoptotic pathway, P21-mediated apoptotic pathway, and other reported pathways. In conclusion, we summarized the experiment-based molecular mechanisms and regulatory networks of NPs for EC. Hopefully, this review focuses on the importance of natural medicines in treating EC and provides a foundation for developing potential anti-EC drugs from natural therapies. There are more and more studies about NPs, and the depth of the research is increasing (Kirchmair, 2020; Naeem et al., 2022). NPs and their biological activities are currently a subject of great interest in the pharmaceutical (Ekiert and Szopa, 2020). Hopefully, the information presented in this review might be significant for further preclinical and clinical investigation.
