# Supplementary material for: Natural products modulate cell apoptosis: a promising way for treating endometrial cancer
Source: Front Pharmacol. 2023 Jun 8;14:1209412. doi: 10.3389/fphar.2023.1209412 (PMC10285317; doi:10.3389/fphar.2023.1209412)
Supplement: Supplementary file 1 [file DataSheet1.docx]

Supplementary Material

Natural Products Modulate Cell Apoptosis: A Promising Way for Treating Endometrial Cancer

Xin Zhou^1†^, Yiwei Zeng^1†^, Runcheng Zheng^2^, Yuemei Wang^1^, Tao Li^1^, Shanshan Song^2^, Su Zhang^1^, Jinzhu Huang^3,4*^, Yulan Ren^2*^

*** Correspondence:**

Prof. Huang Jin-zhu: huangjinzhu@cdutcm.edu.cn

Prof. REN Yu-lan: renxg2468@163.com

# Supplementary Figures and Tables

## Supplementary Figures


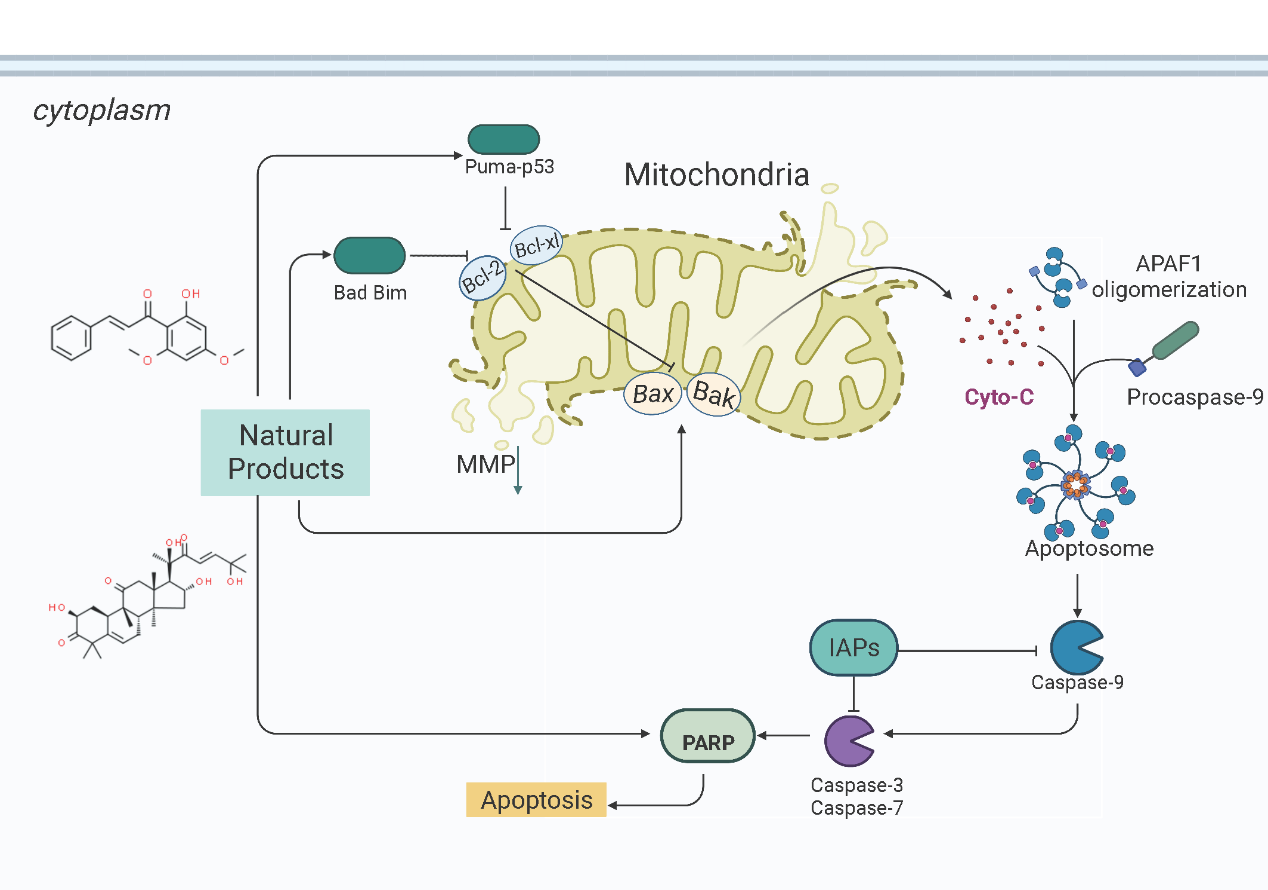


**Supplementary Figure 1.** Natural products modulate apoptosis of ECCs through mitochondria-dependent pathway.


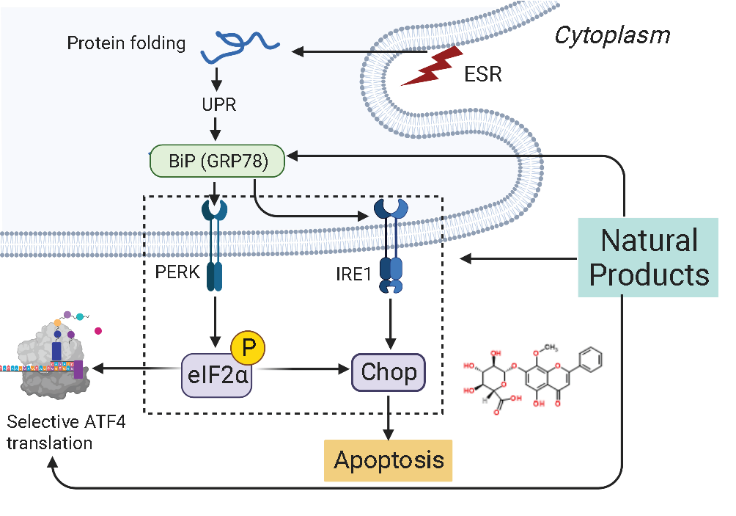


**Supplementary Figure 2.** Natural products modulate apoptosis of ECCs through ERS-mediated pathway.

**
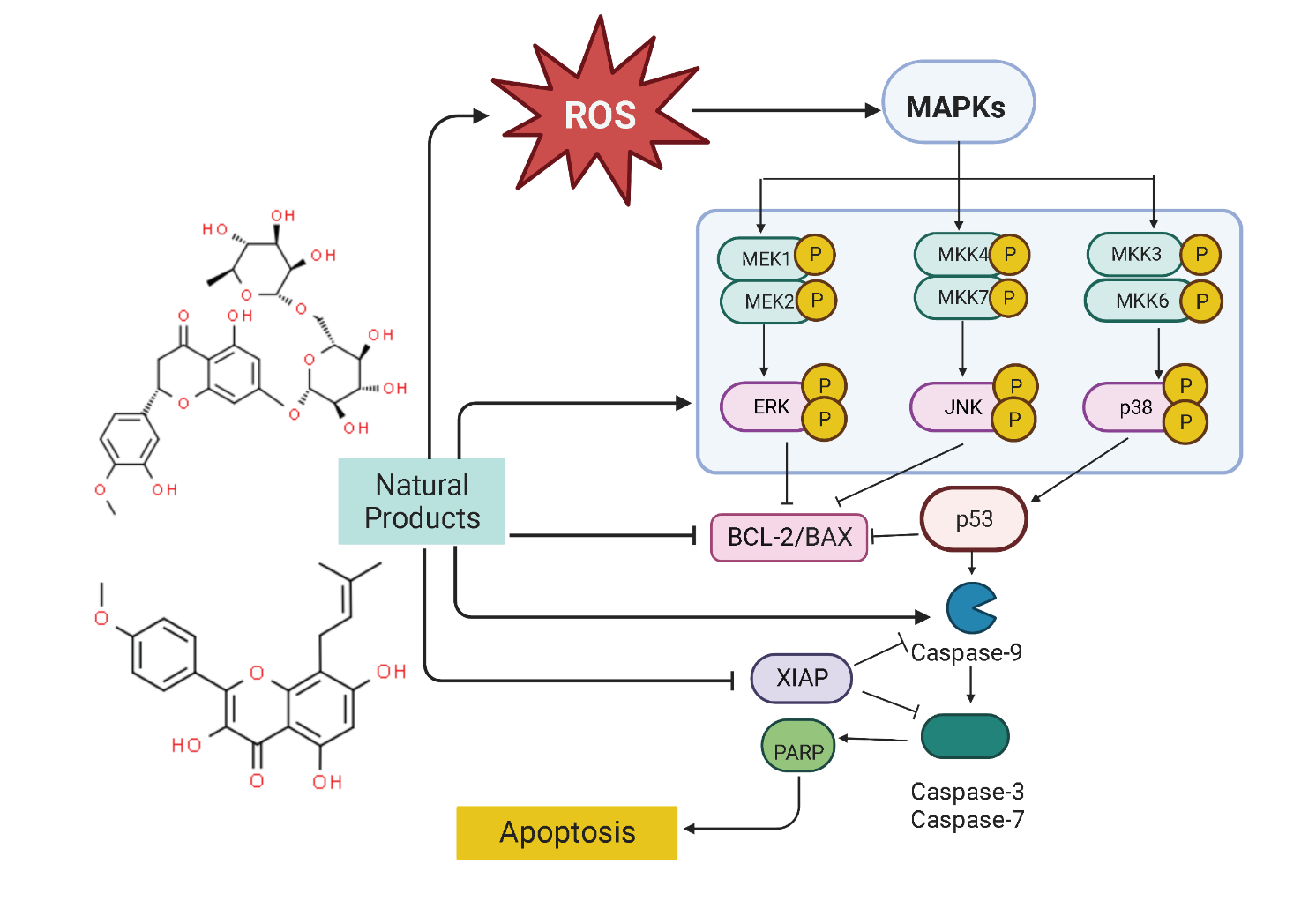
**

**Supplementary Figure 3.** Natural products modulate apoptosis of ECCs through MAPK mediated pathway.


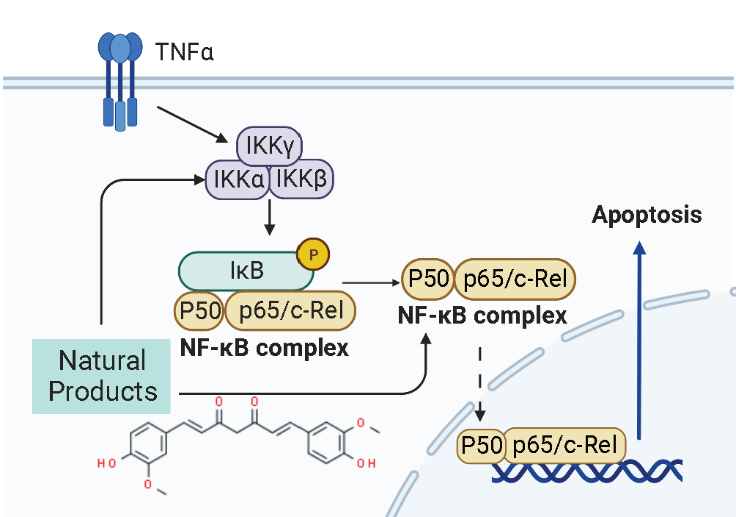


**Supplementary Figure 4.** Natural products modulate apoptosis of ECCs through NF-κB mediated pathway.


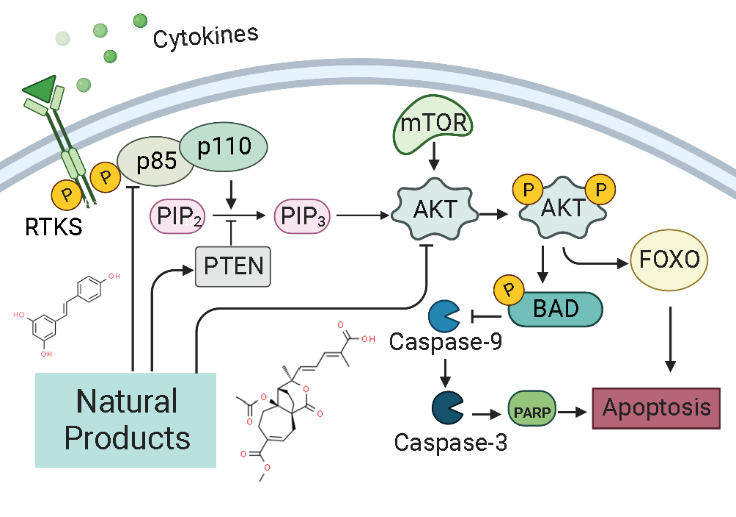


**Supplementary Figure 5.** Natural products modulate apoptosis of ECCs through PI3K/AKT/mTOR pathway.


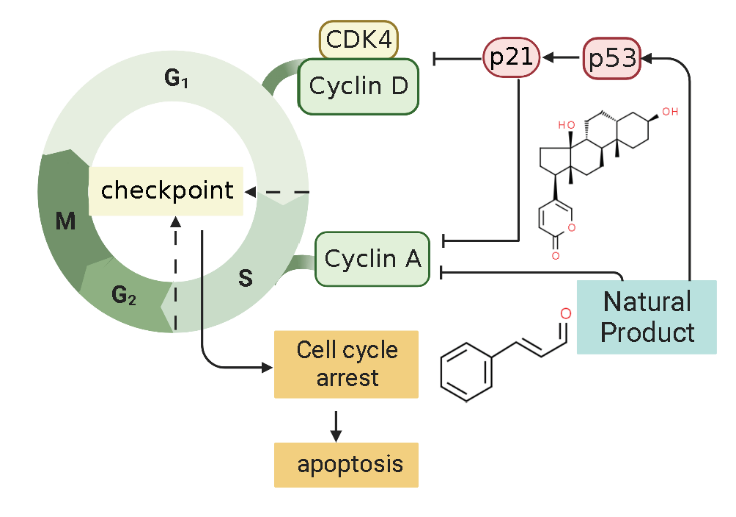


**Supplementary Figure 6.** Natural products modulate apoptosis of ECCs through P21-mediated pathway.

## Supplementary Tables

In almost all cases, detailed information of natural products and their potential effects with mechanisms on modulating apoptosis in EC is illustrated in Table 1, and the chemical structures of isolated metabolites are summarized in Table 2.

### Table 1. Potential effects and mechanisms of natural products on modulating apoptosis in EC

In almost all cases, detailed information of natural products and their potential effects with mechanisms on modulating apoptosis in EC is illustrated in Tables 1, and detailed information and chemical structures of natural products are summarized in Table 2.

### Table 1. Potential effects and mechanisms of natural products on modulating apoptosis in EC.

| **Potential pathways** | **Detailed mechanisms** | **Extracts/monomers (dose/concentration)** | **Cell/Animal model** | **Related targets** | **Refs** |
| --- | --- | --- | --- | --- | --- |
| **Mitochondria-dependent pathway** | Up-regulating caspase-9, -3; Down-regulating bcl-2 | Tian-Long compound (0.05%-0.5%) | Ishikawa cell | Caspase-9, -3, bcl-2 | (Li et al., 2009) |
|  | Increasing p53 phosphorylation; Decreasing bcl-2; | SDGE (0.025-12.50μg/ml) | Ishikawa, ECC-1 cells | p53, bcl-2 | (Liu et al., 2012b) |
|  | Up-regulating bad, bak, bax; Up-regulating bcl-2, bcl-xL, caspase-9, -3, -8 | SOE (50-150μg/ml) | RL95-2 cell | bad, bak, bax, bcl-2, bcl-xL, caspase-9, -3, -8 | (Chang et al., 2014) |
|  | Up-regulating caspase-3, bax; Down-regulating bcl-2 | Zedoary Turmeric Oil (120-960mg/L) | HEC-1B | caspase-3, bax, bcl-2 | (Li et al., 2021) |
|  | Increasing DR5, bim, PUMA; Decreasing survivin | Flavokawain B (1.1-8.8μM) | SK-LMS-1, ECC-1, T-HESC cells | DR5, bim, p53, survivin | (Eskander et al., 2012) |
|  | Incuding Ca2+ influx; Down-regulating bcl-2; Up-regulating bax, caspase-3, -9 | Hyperin (0-500 μM) | RL95-2 cell | Bcl-2, bax, caspase-3, -9 | (Li et al., 2012) |
|  | Decreasing bcl-2, bcl-xL; Increasing caspase-3, -9, PARP | Cucurbitacin D (0.5-4μM) | Ishikawa, HHUA, HEC59 | Bcl-2, bax, caspase-3, -9, PARP | (Ishii et al., 2013) |
|  | Decreasing bcl-2; Increasing p53, caspase-9, -3 | Triptolide (10-320 nM) | HEC-1B cell | Bcl-2, p53, caspase-9, -3 | (Wang et al., 2014) |
|  | Increasing ROS, caspase-9, -8, -3, cyto-c | α-terthienylmethanol (0-2μM) | HEC-1A, Ishikawa cells | ROS, caspase-9, -8, -3, cyto-c | (Lee et al., 2015) |
|  | Decreasing bcl-2; Increasing caspase-3, PARP | Ginsenoside Rh2 (20, 40μM) | Ishikawa, HEC-1A | Bcl-2, caspase-3, PARP | (Kim et al., 2017b) |
|  | Up-regulating caspase-3, bax; Down-regulating bcl-2; Increasing ROS, PARP, p-ERK1/2 | Hinokitiol (1-50μM) | Ishikawa, HEC-1A, KLE cells | Caspase-3, bax, bcl-2, PARP, ERK | (Chen et al., 2021) |
|  | Increasing cyto-c, caspase-3, -9, bax, bim; Decreasing bcl-xL XIAP, survivin | Curcusone C (0.1nM-100μM) | HEC-1A, hESCs | Cyto-c, caspase-3, -9, bax, bim, bcl-xL XIAP, survivin | (An et al., 2021) |
| **ERS mediated stress** | Activating GPR78; Increasing CHOP | Realgar quantum dots (0-30μg/ml) | JEC cells | GPR78, CHOP | (Li et al., 2009) |
|  | Increasing Ca2+ influx, caspase-3, -7, CHOP, PARP | Cannabinoids (0.01-25μM) | Ishikawa, Hec50co | Caspase-3, -7, CHOP, PARP | (Fonseca et al., 2018) |
|  | Increasing PERK, p-eIF2a, ATF4; Activating Hippo signaling pathway | Wogonoside (50μM, 80mg/kg) | Ishikawa  BALB/c-nu mice | PERK, p-eIF2a, ATF4, Hippo | (Chen et al., 2019) |
|  | Up-regulating caspase-3, PARP, JNK, p38; Down-regulating ERK; Activating Akt | ProEGCG (20, 40, 60μM) | AN3 CA, RL95–2 cells | Caspase-3, JNK, p38, ERK,Akt | (Man et al., 2020) |
| **MAPK mediated pathway** | Activating ERK, JNK  Up-regulating caspase-7, -8, -9. -3; Down-regulating Bid, XIAP | Ellipticine (1-10μM) | RL95-2 cell | ERK, JNK, caspase-7, -8, -9, -3, bid, XIAP, AIF, cyt-c | (Kim et al., 2011) |
|  | Increasing bax, ERK1/2; Decreasing bcl-2 | Icaritin (0-10μM) | HeC-1A | ERK, bax, bcl-2 | (Tong et al., 2011) |
|  | Decreasing p-ERK; Increasing caspase-3 | Annonacin (0.2-100μg/ml) | ECCs cells | ERK, caspase-3 | (Chung et al., 2017) |
|  | Up-regulating caspase-3, bax, bik; Down regulating bcl-2, ESR1 | Hesperidin (5-50μM) | ECC-1 cells | Caspase-3, bax, bik, blc-2, ESR1 | (Cincin et al., 2018) |
|  | Up-regulating caspase-3, bax, p38, ERK, JNK, ROS; Down-regulating bcl-2, Akt | Emodin (1.25, 2.5, 5μM) | KLE cells | Caspase-3, bax, bcl-2, p38, ERK, JNK, Akt | (Jiang et al., 2019) |
|  | Inhibiting ERK1/2 phosphorylation, c-Jun | Curcumin (10-80μM) | Ishikawa | ERK, c-Jun | (Zhang et al., 2019b) |
| **NF-κB mediated pathway** | Decreasing NF-κBp50; Up-regulating IκBα, caspase-3 | Scutellaria baicalensis; Fritillaria cirrhosa (1.5-500μg/ml) | EM-E6/E7/TERT, Ishikawa, HEC-1B cells | NF-κBp50, IκBα, caspase-3 | (Kavandi et al., 2015) |
|  | Inhibiting NF-κB; Down-regulating caspase-3 | Curcumin (0-150μM) | Ishikawa, HEC-1 | NF-κB, caspase-3 | (Xu et al., 2018) |
|  | Inhibiting VEGF/PI3K/Akt pathway | Panaxnotoginsengsaponins (50-200μg/ml) | Ishikawa, HEC-1A cells | VEGF, Akt | (Tan et al., 2016) |
| **P13K/Akt/mTOR pathway** | Decreasing p-AKT; Up-regulating caspase-3;  Regulating Akt/mTOR pathway | Resveratrol (0.1, 100μg); (25-200μmol) | HeLa, Hec-1A, KLE, RL95-2, Ishikawa and EN1078D cells | p-AKT, caspase-3, mTOR, p38-AMPK | (Sexton et al., 2006) (Xu et al., 2020) |
|  | Decreasing p-AKT, p-ERK1/2; Increasing caspase-3 | Pseudolaric acid B (0.5-10μmol/l) | Ishikawa cells | AKT, ERK, caspase-3 | (Wang et al., 2017) |
|  | Modulating miR-106b/PTEN/AKT/mTOR pathway; Up-regulating caspase-3, bax; Down- regulating bcl-2 | Shikonin ((10-20μM; 0.3-0.7μg/ml) | Ishikawa, HEC-1A, KLE, RL95-2 cells | miR-106b, PTEN, AKT, mTOR, caspase-3, bax, bcl-2 | (Yin, 2016; Huang and Hu, 2018) |
|  | Increasing bax, Decreasing bcl-2, p-mTOR, p-Akt, p-P13K | Kaempferol (0-20μM) | MFE-280 | Bax, bcl-2, P13K, Akt, mTOR | (Lei et al., 2019) |
|  | Up-regulating bax; Down-regulating bcl-2, P13K, Akt, mTOR | Amygdalin (8-128mg/L) | EECs, RL95-2, HEC-1B | Bax, bcl-2, P13K, Akt, mTOR | (Ye et al., 2020) |
|  | Decreasing P13K, Akt, mTOR  Up-regulating bax, bak, bad, cyto-c, caspase-3, -9; Down-regulating bcl-xL | Asparanin A (6-18μM) | Ishikawa cells  Female BALB/c-nu mice | P13K, Akt, mTOR, bax, bak, bad, cyto-c, caspase-3, -9, bcl-xL | (Zhang et al., 2020) |
|  | Up-regulating bax, caspase-3, -9, PARP, PETN; Down-regulating P13K, Akt | Osthole (25-200μM) | EC-KLE, Ishikawa cells | P13K, Akt, PETN, bax, caspase-3, -9, PARP | (Liang et al., 2021) |
| **p21-mediated pathway** | Regulating p53-independent pathway | Psammaplin A (1-10μg/ml) | Ishikawa cells | p21^WAF1^, p53 | (Ahn et al., 2008) |
|  | Down-regulating cyclin A, cyclin D3, bcl-2 and bcl-xL; Up-regulating p21^WAF1^, caspase-9 | Bufalin (1ng/ml) | Ishikawa, HHUS, HEC-1B, NHEEC cells | Cyclin A, cyclin D3, bcl-2 and bcl-xL, p21^WAF1^, caspase-9 | (Takai et al., 2008) |
|  | Up-regulating p21; Down-regulating CDK4, MMP2, MMP9 | Cinnamaldehyde (3.75, 7.5, 15μg/ml) | Ishikawa cells | p21, CDK4, MMP2, MMP9 | (Dong and Li, 2021) |
| **Other** | Not concluded | Rice bran fraction (100, 200, 300μg/ml) | Sawano cell | Not concluded | (Fan et al., 2000) |
|  | Up-regulating BAG3, caspase-4, -5 | PCAE (0-4 mg/ml) | Ishikawa cells | BAG3, caspase-4, -5 | (Tsai et al., 2015) |
|  | Increasing ROS, bax; Decreasing bcl-2; Inhibiting pSTAT1, pSTAT2, pJAK1, pJAK2 | Tanshinone l (0-40μM) | HEC-1-A cells | Bax, bcl-2, STAT, JAK | (Li et al., 2018) |
|  | Up-regulating caspase-3, -7, PARP | Isoliquiritigenin (5-100μM) | HEC-1-A, Ishikawa | Caspase-3, -7, PARP | (Wu et al., 2016a) |
|  | Inhibiting STAT3; Decreasing bcl-2, survivin | Silibinin (100, 150, 200μM) | Ishikawa, RL-952 | STAT3, bcl-2, survivin | (Shi et al., 2019) |
|  | Increasing miR-424 caspase-3, -9; Decreasing CPEB2 | Osthole (50, 100, 200μM) | Ishikawa, KLE | miR-424, CPEB2, caspase-3, -9 | (Lu et al., 2020) |
|  | Increasing caspase-3 | Gallic Acid (5-100μg/ml) | Ishikawa cells | Caspase-3 | (Bulbul et al., 2021) |
|  | Down-regulating XIAP, bcl-xL, pAKT via hnRNPA1 | Esculetin (0-120μM) | HEC-1B, Ishikawa cells | hnRNPA1, XIAP, bcl-xL, pAKT | (Jiang et al., 2021b) |
|  | Up-regulating caspase-3 | Silymarin (6μg/ml) | Ishikawa cells | caspase-3 | (Hua et al., 2019) |

DR5, death receptor 5; PUMA, p53 Upregulated Modulator of Apoptosis; SDGE, Steam Distilled Extract of Ginger; SOE, Siegesbeckia orientalis ethanol extract; CHOP, C/EBP homologous protein; GPR78, G-protein coupled receptor 78; ERK, extracellular-signal-regulated kinase; JNK, c-Jun N-terminal kinase; ProEGCG, prodrug of (−)-epigallocatechin-3-gallate; AMPK, AMP-activated protein kinase; XIAP, X-linked inhibitor of apoptosis protein; AIF, apoptosis inducing factor; ESR1, estrogen receptor I; Cyto-c, cytochrome-c; β-HIVS, β-Hydroxyisovalerylshikonin; PARP, poly-ADP ribose polymerase; BAG3, BCL-associated athanogene 3; VEGF, vascular endothelial growth factor; PCAE, Pogostemon cablin Aqueous Extract

### Table 2. Detailed information and chemical structures of natural products.

| **Monomers** | **Origin** | **Systematic name** | **Chemical structures** |
| --- | --- | --- | --- |
| Flavokawain B | Piper methysticum | (2E)-1-(2-Hydroxy-4,6-dimethoxyphenyl)-3-phenyl-2-propen-1-one | 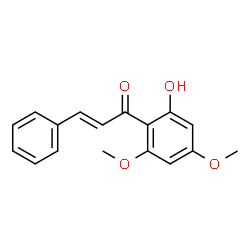 |
| Hyperin | [Rhododendron dauricum L.](https://mpns.science.kew.org/mpns-portal/plantDetail?plantId=2419620&query=rhododendron+leaf&filter=&fuzzy=false&nameType=all&dbs=wcsCmp) | 2-(3,4-Dihydroxyphenyl)-5,7-dihydroxy-4-oxo-4H-chromen-3-yl β-D-galactopyranoside | 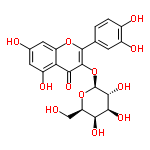 |
| Cucurbitacin D | Pyrus communis subsp. communis | (2S,4R,9β,16α,23E)-2,16,20,25-Tetrahydroxy-9,10,14-trimethyl-4,9-cyclo-9,10-secocholesta-5,23-diene-1,11,22-trione | 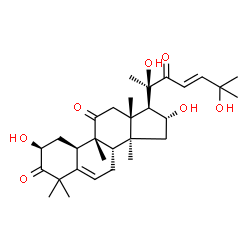 |
| Triptolide | [Tripterygium wilfordii Hook.f.](https://mpns.science.kew.org/mpns-portal/plantDetail?plantId=2431667&query=Tripterygium+wilfordii+Hook.+F&filter=&fuzzy=false&nameType=all&dbs=wcsCmp) | (3bS,4aS,5aS,6R,6aR,7aS,7bS,8aS,8bS)-6-Hydroxy-6a-isopropyl-8b-methyl-3b,4,4a,6,6a,7a,7b,8b,9,10-decahydrotrisoxireno[6,7:8a,9:4b,5]phenanthro[1,2-c]furan-1(3H)-one | 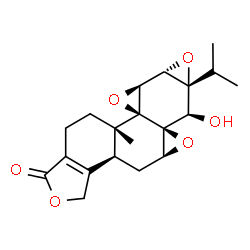 |
| alpha-Terthienylmethanol | [Eclipta prostrata (L.) L.](https://mpns.science.kew.org/mpns-portal/plantDetail?plantId=2901074&query=Eclipta+prostrata&filter=&fuzzy=false&nameType=all&dbs=wcsCmp) | 2,2':5',2''-Terthiophen-5-ylmethanol | 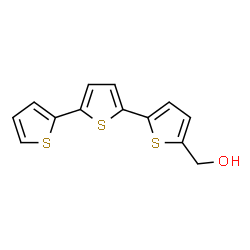 |
| Ginsenoside Rh2 | Panax ginseng C.A.Mey. | (3β,12β)-12,20-Dihydroxydammar-24-en-3-yl β-D-glucopyranoside | 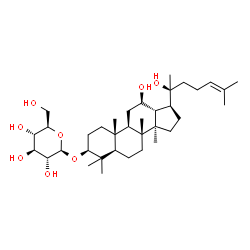 |
| Hinokitiol | Chamaecyparis obtusa var. formosana (Hayata) Hayata | 2-Hydroxy-4-isopropyl-2,4,6-cycloheptatrien-1-one | 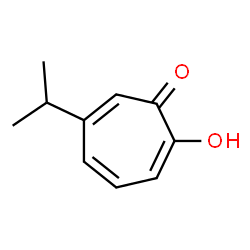 |
| Curcusone C | Jatropha curcas L. | (2S,6aS)-2-Hydroxy-7-isopropenyl-2,5-dimethyl-10-methylene-2,3,6a,7,8,9,10,10a-octahydrobenzo[e]azulene-1,4-dione | 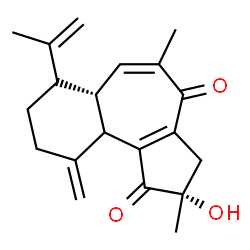 |
| Wogonoside | Scutellaria baicalensis Georgi | 5-Hydroxy-8-methoxy-4-oxo-2-phenyl-4H-chromen-7-yl β-D-glucopyranosiduronic acid | 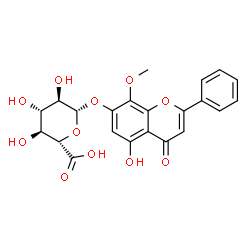 |
| Ellipticine | Ochrosia elliptica Labill. | 5,11-Dimethyl-6H-pyrido[4,3-b]carbazole | 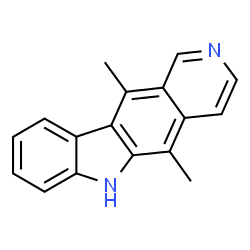 |
| Icaritin | Epimedium brevicornu Maxim. | 3,5,7-Trihydroxy-2-(4-methoxyphenyl)-8-(3-methyl-2-buten-1-yl)-4H-chromen-4-one | 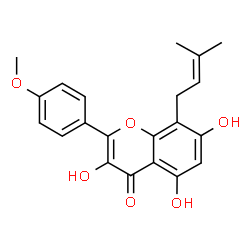 |
| Annonacin | Annona muricata L. | (5S)-5-Methyl-3-[(2R,8R,13R)-2,8,13-trihydroxy-13-{(2R,5R)-5-[(1R)-1-hydroxytridecyl]tetrahydro-2-furanyl}tridecyl]-2(5H)-furanone | 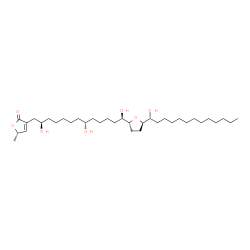 |
| Hesperidin | Citrus × aurantium L. | (2S)-5-Hydroxy-2-(3-hydroxy-4-methoxyphenyl)-4-oxo-3,4-dihydro-2H-chromen-7-yl 6-O-(6-deoxy-α-L-mannopyranosyl)-β-D-glucopyranoside | 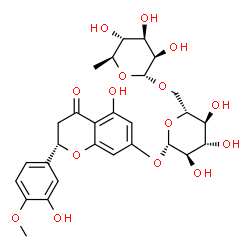 |
| Emodin | Rheum palmatum L. | 1,3,8-Trihydroxy-6-methyl-9,10-anthraquinone | 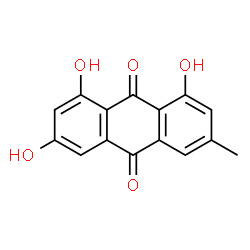 |
| Curcumin | Curcuma longa L. | (1Z,6Z)-1,7-Bis(4-hydroxy-3-methoxyphenyl)-1,6-heptadiene-3,5-dione | 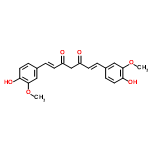 |
| Scutellaria baicalensis | Scutellaria baicalensis Georgi | 3-(9,9-Dimethyl-10(9H)-acridinyl)-N,N-dimethyl-1-propanamine | 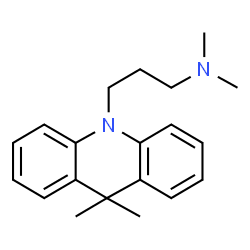 |
| Resveratrol | Red wine | 5-[(E)-2-(4-Hydroxyphenyl)vinyl]-1,3-benzenediol | 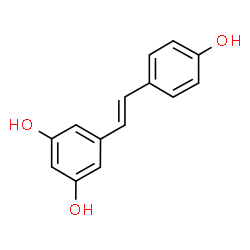 |
| Pseudolaric acid B | Larix kaempferi (Lamb.) Carrière | (2E,4E)-5-[(1R,7S,8S,9R)-7-Acetoxy-4-(methoxycarbonyl)-9-methyl-11-oxo-10-oxatricyclo[6.3.2.01,7]tridec-3-en-9-yl]-2-methyl-2,4-pentadienoic acid | 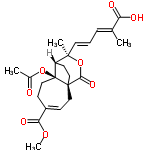 |
| Shikonin | Lithospermum erythrorhizon Siebold & Zucc. | 5,8-Dihydroxy-2-[(1R)-1-hydroxy-4-methyl-3-penten-1-yl]-1,4-naphthoquinone | 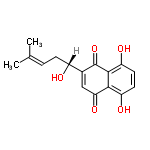 |
| Kaempferol | Kaempferia galanga L. | 3,5,7-Trihydroxy-2-(4-hydroxyphenyl)-4H-chromen-4-one | 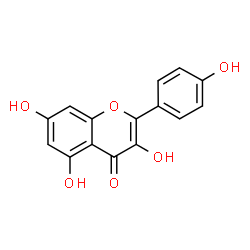 |
| Amygdalin | Prunus amygdalus Batsch | (2R)-{[6-O-(β-D-Glucopyranosyl)-β-D-glucopyranosyl]oxy}(phenyl)acetonitrile | 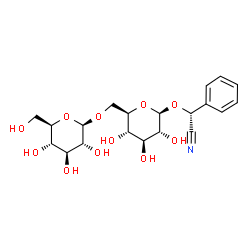 |
| Asparanin A | Asparagus officinalis L. | (3β,5β,25S)-Spirostan-3-yl 2-O-β-D-glucopyranosyl-β-D-glucopyranoside | 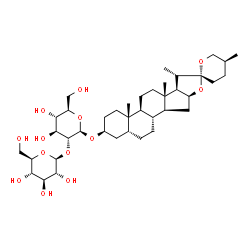 |
| Osthole | Cnidium monnieri (L.) Cusson | 7-Methoxy-8-(3-methyl-2-buten-1-yl)-2H-chromen-2-one | 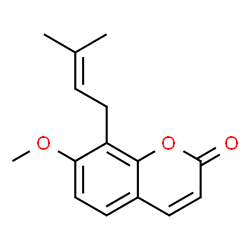 |
| Isoliquiritigenin | Glycyrrhiza glabra L. | (2E)-1-(2,4-Dihydroxyphenyl)-3-(4-hydroxyphenyl)-2-propen-1-one | 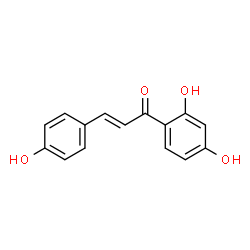 |
| Esculetin | Fraxinus chinensis subsp. rhynchophylla (Hance) A.E.Murray | 6,7-Dihydroxy-2H-chromen-2-one | 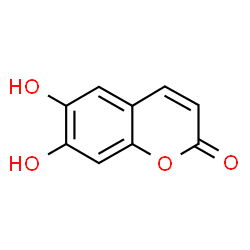 |
| Silymarin | Silybum marianum (L.) Gaertn. | 3,5,7-trihydroxy-2-[3-(4-hydroxy-3-methoxyphenyl)-2-(hydroxymethyl)-2,3-dihydro-1,4-benzodioxin-6-yl]-2,3-dihydrochromen-4-one | 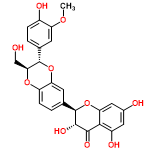 |
